# Animal Disease Surveillance in the 21st Century: Applications and Robustness of Phylodynamic Methods in Recent U.S. Human-Like H3 Swine Influenza Outbreaks

**DOI:** 10.3389/fvets.2020.00176

**Published:** 2020-04-21

**Authors:** Moh A. Alkhamis, Chong Li, Montserrat Torremorell

**Affiliations:** ^1^Department of Epidemiology and Biostatistics, Faculty of Public Health, Health Sciences Center, Kuwait University, Kuwait City, Kuwait; ^2^Department of Veterinary Population Medicine, College of Veterinary Medicine, University of Minnesota, St. Paul, MN, United States

**Keywords:** human-like H3, swine influenza, evolutionary epidemiology, phylodynamics, phylogeography, disease surveillance

## Abstract

Emerging and endemic animal viral diseases continue to impose substantial impacts on animal and human health. Most current and past molecular surveillance studies of animal diseases investigated spatio-temporal and evolutionary dynamics of the viruses in a disjointed analytical framework, ignoring many uncertainties and made joint conclusions from both analytical approaches. Phylodynamic methods offer a uniquely integrated platform capable of inferring complex epidemiological and evolutionary processes from the phylogeny of viruses in populations using a single Bayesian statistical framework. In this study, we reviewed and outlined basic concepts and aspects of phylodynamic methods and attempted to summarize essential components of the methodology in one analytical pipeline to facilitate the proper use of the methods by animal health researchers. Also, we challenged the robustness of the posterior evolutionary parameters, inferred by the commonly used phylodynamic models, using hemagglutinin (HA) and polymerase basic 2 (PB2) segments of the currently circulating human-like H3 swine influenza (SI) viruses isolated in the United States and multiple priors. Subsequently, we compared similarities and differences between the posterior parameters inferred from sequence data using multiple phylodynamic models. Our suggested phylodynamic approach attempts to reduce the impact of its inherent limitations to offer less biased and biologically plausible inferences about the pathogen evolutionary characteristics to properly guide intervention activities. We also pinpointed requirements and challenges for integrating phylodynamic methods in routine animal disease surveillance activities.

## Introduction

In the past few decades, genetic analysis of rapidly evolving pathogens has become an integral part of animal disease surveillance systems worldwide (1–4). Most current and past molecular surveillance studies of animal disease pathogens of both public health and economical importance such as influenza (5–7), foot-and-mouth disease (FMD) (8–10), and porcine reproductive and respiratory syndrome (PRRS) (11–13) viruses are dependent on classical epidemiological and phylogenetic methods. These studies or surveillance systems used classical phylogenetic methods, including parsimony, neighbor-joining, or maximum likelihood (ML) approaches to either genotype novel emerging strains, classify viral lineages, or assess tree topologies to distinguish between novel and emerging strains (6, 7, 13). In addition, classical phylogenetic approaches were used to assess correlations between the similarities of nucleotide sequences and related epidemiological characteristics, while ignoring uncertainties associated with estimates of phylogenetic relationships, host, temporal, and spatial factors (7, 10, 11, 14). Furthermore, they investigated spatio-temporal and evolutionary dynamics of the virus isolates in a disjointed analytical framework and made joint conclusions from both analytical approaches (7, 10, 11, 14). Therefore, many of the past and current molecular surveillance studies of animal diseases have ignored that epidemiological and evolutionary dynamics of rapidly evolving viruses occur on approximately the same time-scale (15). Thus, studying them in a unified analytical framework will refine their interpretations and limit biased conclusions to subsequently improving the related molecular surveillance activities. Classical phylogenetic approaches are not capable of accounting for the uncertainties in evolutionary processes of rapidly evolving pathogens or integrating related epidemiological features into their phylogeny, which is an important advantage of Bayesian phylodynamic methods.

The Bayesian phylodynamic methods were borrowed from the field of evolutionary biology and have become a powerful tool for exploring the evolutionary epidemiology of infectious pathogens (14–17). During the last two decades, the rapid growth of pathogens' genetic data and computational resources increased the applications of phylodynamic methods in animal and human disease surveillance (17). These methods are capable of accounting for uncertainties, and uniquely integrate complex epidemiological and evolutionary processes in populations using a single Bayesian statistical framework (18, 19). This framework handles the parameters of the phylodynamic model as random variables, in which each parameter is set by a specified prior probability distribution (and a parallel inferred posterior probability distribution). Therefore, this innovative quantitative integration improved disease investigation by answering novel epidemiological questions about the evolutionary history, spatiotemporal origins, within and between-host transmission, and environmental risk factors for rapidly evolving pathogens (17). In fact, during the last decade, phylodynamic models have become well-established tools for studying the evolution of animal viral diseases specially influenza (20), FMD (17), and PRRS (21). Besides, several studies advocated for the integration of phylodynamic methods in the routinely molecular surveillance pipelines of animal diseases with the objectives of reclassifying viral genotypes, distinguishing between emerging and endemic viral strains, and selecting proper vaccine strains (17, 21–23). These approaches will provide a robust platform for guiding the allocation of resources within a surveillance system, for example, targeting emerging strains with higher evolutionary rates or hosts at high risk of generating new strains, which subsequently will reduce the economic costs of sampling, control, and prevention activities. Phylodynamic methods are implemented in many open-source statistical software packages, while the most popular user-friendly software package is formally known as Bayesian evolutionary analysis by sampling tree (BEAST) (24).

While past studies illustrated the great potential of phylodynamic tools, the methods are sensitive to the density and coverage of sequence sampling, selection of genetic regions, quality and quantity of the associated surveillance data, and prior selection for the evolutionary parameters (15, 25, 26). These limitations may result in biased posterior inferences, which subsequently lead to inaccurate or biological implausible conclusions about the evolutionary epidemiology of the pathogen under study (e.g., false divergence time or geographical origins). That said, most phylogenetic studies suffer from these inherent limitations. However, setting a thorough phylodynamic analytical pipeline, while acknowledging these limitations, can reduce their impact on the resulting posterior inferences and their related conclusions. Unfortunately, many published phylodynamic studies ignored such limitations, particularly in their analytical approach, in which they used simple naïve priors for their evolutionary parameters while ignoring the underlying assumptions for these priors (27–31). For example, prior selection should adhere to the assumption that different pathogens have unique evolutionary characteristics (14), and therefore, using the same simple prior on different pathogens will likely lead to the conclusion that such pathogens behaved similarly during their evolutionary history. Also, these studies ignored the impact of selecting different prior models on their posterior evolutionary inferences of the pathogen under study (26, 32). For example, the use of different prior models often leads to different conclusions about the geographical origins of the pathogen under study, and hence, Bayesian model selection is a critical step in phylodynamic analysis pipelines (25, 33).

There are many studies in the published literature comparing the results of phylodynamic models inferred from different gene segments or evolutionary parameters' priors (34–36). However, few studies raised concerns about the sensitivity of the results to the choice of different evolutionary models (20, 26) as well as suggested a focused phylodynamic analytical pipeline for animal disease molecular surveillance (37). Here, we demonstrate the basic principles for building a phylodynamic analytical pipeline, illustrate examples on the impact of gene segment and prior selection on the posterior evolutionary inferences, and highlight the prospects of the methods in improving animal disease surveillance. We selected a publicly available dataset compromising of 352 full genome sequences for human-like H3 swine SI collected as part of the United States Department of Agriculture influenza surveillance system between 2015 and 2018 as a working example. We provided a detailed description of a classical phylodynamic analytical pipeline encompassing both demographic and discrete phylogeographic reconstruction of the human-like H3 virus using BEAST. Our phylodynamic analyses included comparisons between commonly inferred evolutionary posterior parameters (e.g., substitution rate/site/year, divergence times, phylogeographic root state posterior probabilities, significant dispersal route between states) under different combinations of node–age and branch rate prior models. Furthermore, we extended this analytical pipeline into comparing posterior parameters inferred from HA and PB2 gene segments. Interpretation of the resulting posterior inferences under different scenarios, described above, has been discussed in detail, and we highlighted examples of their misuse in past phylodynamic studies. Our results identified the prospects and limitations of the presented phylodynamic pipeline in the context of animal disease surveillance on regional and global scales. Furthermore, our results provide researchers and stakeholders of the swine industry in the United States valuable insights on decisions related to the sampling and sequencing of the influenza virus genome when conducting future phylodynamic studies and improving the design of currently implemented surveillance systems.

## Bayesian Phylodynamic Statistical Framework

**Figure 1:**
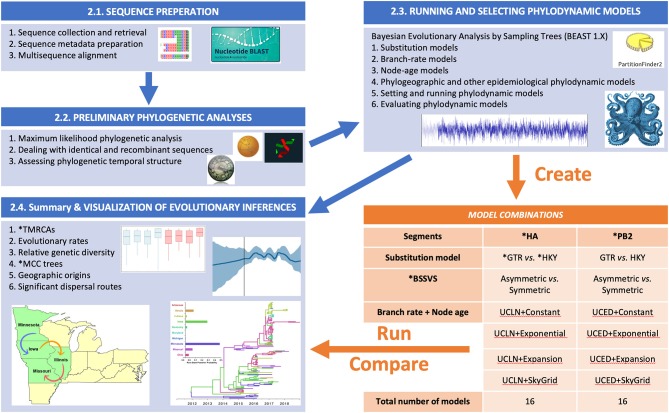
Flow chart showing the steps for our Bayesian phylodynamic statistical framework. Blue boxes summarize the methodological steps for the statistical framework described in the BAYESIAN PHYLODYNAMIC STATISTICAL FRAMEWORK section of the main text (Sequence Preparation section to Summary and Visualization of Evolutionary Inferences section). The orange table indicates the generated models for swine influenza example data described in Section Worked Example: Evolutionary Dynamics of Swine Influenza in the United States Between 2015 and 2018. ML, maximum likelihood; RDP, Recombination Detection Program; ESS, effect sample size; BF, Bayes factor; PS, path sampling; SS, stepping stone; HA, hemagglutinin; PB2, polymerase basic 2; GTR, general time-reversable; HKY, Hasegawa, Kishino, and Yano; BSSVS, Bayesian Stochastic Search Variable Selection; TMRCAs, Time to the Most Recent Common Ancestor; MCC, Maximum Clade Credibility.

The summary flow chart of our phylodynamic analytical pipeline is presented in Figure 1. This Bayesian statistical framework is popular and well-established for studying rapidly evolving pathogens as described elsewhere (37–39). The pipeline is divided into five steps (Figure 1), in which two steps are dedicated to sequence preparation and curation of relevant viral lineages, while the following three steps are dedicated for phylodynamic analyses of the subsequently selected lineages.

### Sequence Preparation

#### Sequence Collection and Retrieval

A critical step for a sound phylodynamic analysis is sequence preparation. This step can take two directions, depending on the study design and the objectives of the analysis. The first direction involves primary data analyses of novel sequences, in which they are either part of a designed study to identify the evolutionary characteristics of newly emerging viral strains (27, 37, 39) or part of an ongoing active surveillance program (40). This direction usually includes the collection and sequencing of novel viral isolates from ongoing outbreaks. The second direction involves secondary data analyses of sequence collections published in publicly available genomic databases such as the Genbank, to mainly explore the evolutionary history of specific pathogens either on regional or global scales (38, 41, 42). Secondary sequence analysis can either target all available viral isolates or specific well-defined lineages (i.e., monophyletic clades) (38, 41, 42). To reduce the impact of sampling bias on the results of a phylodynamic analyses, it is essential to ensure the representativeness of the viral isolates under study to the available sequences data on both temporal and spatial scales. This step is most important for primary sequence analyses, in which the dataset under study needs to cover all close relatives of novel viral isolates published elsewhere. Retrieving and combining relatives of novel viral isolates in a single dataset will warrant a proper inference of representative phylogenetic relationships of a tree topology based on all available related sequences. As on many occasions, novel sequences might belong to different distinct viral lineages published elsewhere (39, 43). The basic local alignment search tool (BLAST; https://blast.ncbi.nlm.nih.gov/Blast.cgi) is the most popular tool for retrieving relatives of novel sequences (Figure 1). Finally, the retrieval process should include complete and near-complete sequences to avoid distorting the phylogenetic relationships between the novel and the related isolates.

#### Sequence Metadata Preparation

The integration of a pathogen's epidemiological characteristics into its inferred phylogeny is the ultimate justification for the preference of the phylodynamic approach over the classical phylogenetic methods. Therefore, the thorough preparation of sequence metadata, which includes retrieval of information related to the isolate under study, is another critical step for a sound subsequent phylodynamic analysis. Sequence metadata can be retrieved either from public genomic databases such as the Genbank or from the related published literature. Because phylodynamic methods largely depend on time-stamped data, this step starts with retrieving the data of collection for the viral isolates under study. Thus, viral isolates with no temporal data are typically excluded from the analyses pipeline. Next, the date of collection is converted into BEAST readable format known as fractional years to estimate divergence times. For example, a virus collected on April 14, 2017, is converted into “2017.282” as a fractional year, where “2017” is the year of collection, and “0.282” is the number of days from the beginning of that year till the day for sequence collection divided by the total number of days within a typical year. Additionally, dates can be imported to BEAUTi by a separate text file that include the complete date of sequence collection with explicit separators (e.g., – or /). However, in many instances, the complete date of collection is not available, in which it misses either the exact date or month of collection. Therefore, we can either specify the age of the isolate as the mid-point of the corresponding month or year, respectively. Other epidemiological characteristics such spatial or host information can be prepared in a separate text delaminated format with unique identifiers that link them to the isolates in the sequence dataset. Isolates missing a none-temporal information should be kept in the analyses and are usually labeled with a question mark “?” to represent a missing information. In the context of the phylodynamic field, epidemiological characteristics such as country or host of origin are defined as a discrete trait and are described in more detail in the Running and Selecting Phylodynamic Models section. However, careful selection of these characteristics is recommended to be considered at the beginning of the analyses pipeline as a critical part of the data preparation for the subsequent analyses. Geographical discrete traits can be defined as the country of origin where the pathogen was isolated or can be redefined on smaller or larger spatial scales such as administrative regions within a country (44) or continental scale (32), respectively, depending on the study's hypothesis. Besides the host of origin, other non-spatial discrete traits such as host and environmental attributes can also be defined as discrete traits (45).

#### Multisequence Alignment

Multisequence alignment (MSA) is another primary key step in the data preparation stage of the pathogen's genetic data (Figure 1). It is worth noting that alignment uncertainty, for example, in terms of the choice of alignment algorithm can affect the subsequent phylogenetic inferences, such as tree topology (46). However, the impacts of alignment uncertainties have not been reported with simple pathogens like viruses, mainly when dealing with small gene segments. Therefore, this issue might be considered when dealing with whole genomes or with more complex pathogens like bacteria and fungi, which can be resolved by multiple sequence alignment averaging using different alignment algorithms (47). Common alignment algorithms include CLUSTAL (48), T-coffee (49), and MUSCLE (50), while AliView is a user-friendly graphical interface that can deal with large sequence datasets and integrate multiple alignment algorithms (51). Performing the multisequence alignment using an algorithm, and manually deleting the gaps within the translated alignment, are the most common steps for most phylogenetic studies (51). Also, confirming the reading frame of each gene segment (excluding the 5′UTR) by examining the amino acid translation is another step within the MSA procedure. This step is commonly done, for example, for influenza virus HA and PB2 gene segments, and potentially for segments 7 and 8, to account for the frameshifted M2 and NS2 genes. However, it is worth noting that this step is only important for partitioned nucleotide models, described below.

### Preliminary Phylogenetic Analyses

#### Inferring Preliminary Phylogenetic Trees

Phylodynamic analyses require both time and computational resources, and therefore, conducting exploratory phylogenetic analyses using classical methods is an essential step that will ensure the proper setup of the subsequent phylodynamic models' priors. Classical methods for inferring basic phylogenetic trees (i.e., non-time-stamped trees) include the maximum likelihood (ML) (52), maximum parsimony (MP) (53), and neighbor-joining (54) algorithms. Inferring the basic phylogenetic tree of a sequence dataset will help in the preliminary assessment of the tree's topology in terms of the magnitude of structure across branches, degree of topological (in)congruence between different gene segments, and selection of lineages (in large datasets) for the subsequent phylodynamic analyses. Classical phylogenetic algorithms are implemented in many open-source software packages such as MEGA (55) and RaXML (56).

#### Dealing With Identical and Recombinant Sequences

The rapid spread and transmission of viral diseases during epidemics provide plenty of time for the pathogen to accumulate informative mutations in their genomes (57). Therefore, 100% identical sequences within a dataset dilute such information. Also, retrieved sequence datasets suffer from inherent redundancy due to sampling bias and issues related to the sequencing procedure (58). Hence, removing 100% identical sequences from the dataset under study, will reduce the impact of such redundancies, strengthen the tree structure, and shorten the computational time. Furthermore, if the proportion of 100% identical sequences was substantially large, it will typically lead to weaker evolutionary signals and subsequently poorer phylodynamic model convergence.

Recombination is a natural biological phenomenon of rapidly evolving viruses like influenza and occurs when viral genomes co-infect the same host cell and exchange fragments of their gene segments resulting in new viral strains (59). Ignoring recombination events in a sequence dataset may advisedly bias the inferred posterior phylogenetic relationships and, therefore, must be excluded (60). Recombination events can be detected using the Recombination Detection Program (61). However, recombination events are more often detected in whole genomes than in single-gene segments. Therefore, conducting phylodynamic analyses on whole-genome sequences only will lead to the exclusion of many isolates resulting in a substantially smaller dataset and subsequently biased inferences. Nevertheless, the occurrence of recombination events at the beginning of a novel viral outbreak might be limited.

#### Assessing Phylogenetic Temporal Structure

Assessing the magnitude of temporal structure in the phylogeny of the sequences data collected at different points of time is the final recommended step within the preliminary phylogenetic analyses stage (62). Here, the term “temporal structure” is defined as the measurable difference in terms of nucleotide or amino acid substitution between two genetic sequences sampled at two distinct points of time (63). Therefore, if the sequence data lacks sufficient temporal structure, then proceeding to the phylodynamic analysis may lead to biased posterior estimates and misleading conclusions (62). An interactive regression-based approach is implemented in the TempEst software package (62) to assess the strength of the association between sequences' sampling dates and genetic divergence through time. *R*^2^ values closer to 1 than 0 estimated from a time-stamped ML tree using the root-to-tip genetic distance linear regression indicate a strong temporal structure (62). Finally, TempEst can identify incongruent sequences that are defined as outlier isolates that caused substantially more or less genetic divergence from the tip to the root than one would expect given their sampling date (62). Incongruent sequences usually result from low sequencing quality, alignment errors, laboratory adopted and vaccine strains, as well as natural biological processes such as recombination.

### Running and Selecting Phylodynamic Models

Once the sequence dataset and their metadata are curated (by the past two steps, described above), we provide a variety of choices for selecting and running phylodynamic models depending on the objectives of the study. Steps involving prior specification, simulations, and summarizing posterior inferences are all implemented in the BEAST software package (24).

#### Substitution Models

Large evolutionary distances (i.e., substitution per site) between pairs of sequences caused by multiple substitution events through time can be underestimated when using simple distance measures (e.g., Hamming distance) (64). Hence, the distance correction technique provided by the substitution models can compensate for the underestimation of such large evolutionary distances (64). Phylogenetic tree algorithms such as the ML approach incorporates substitution models that employs continuous-time Markov chain (CTMC) models (52). CTMC models are stochastic methods that take values from a discrete state evolutionary space at random times, which is analogous to a nucleotide or amino acid substitution process, allowing for glimpsing the complete state history over the entire phylogeny where statistical inferences are drawn (52, 64, 65). Out of many available substitution models, the Hasegawa, Kishino, and Yano (HKY) (66) and the general time-reversable (GTR) (52, 67) are the most common models used to infer the phylogeny of rapidly evolving pathogens. Briefly, both substitution models assume a constant rate of evolution and have two major parameters, including a rate matrix (Q) and an equilibrium vector of base frequencies. However, the HKY model rate matrix has two exchangeability parameters, including one transition rate and one transversion rate parameters (66), while the GTR model has a symmetrical substitution rate matrix where all the exchangeability parameters are free (67).

Accommodating the rate variation across sites can be achieved by combining substitution models with site models such as the discrete gamma (Γ) model (68). However, when assuming that the evolution rate is equal to zero, the invariant site (*I*) model is combined with the corresponding substitution model (69). Selection pressure in protein-coding genes of rapidly evolving pathogens, in terms of synonymous to non-synonymous substitutions, usually occurs at high rates (70). This evolutionary phenomenon can affect estimates of divergence time and, therefore, need to be accounted for when selecting a substitution model (71). Partitioning the gene segment into unique codon positions and assigning different substitution and site model combinations can accommodate the differences in the evolutionary dynamics within gene segments of the pathogen under study (70, 72). Different substitution, sites, and codon partitioning models are implemented in many ML software packages as well as in BEAST. However, selecting the most realistic substitution/site model and partitioning scheme for the sequence data can be statistically achieved using either Bayesian Information Criterion (BIC) (73), Akaike Information Criterion (AIC), or the corrected Akaike Information Criterion (AICc) (74, 75). These ML-based statistical methods are well-implemented in both PartitionFinder (76) and jModelTest (77). Yet, a more robust Bayesian method for selecting a site model and an associated substitution model is implemented as an add-on package in BEAST 2.X (78).

#### Branch-Rate Models

Time-calibrated trees are modeled with the genetic differences between sequences through the molecular clock models, which is defined as the clock that occurs after a stochastic waiting time in the context of substitution rate (79). When assuming that the substitution rate across the branches is uniform over the entire tree, then the molecular clock model is defined as strict. However, changes in the rate of evolution of rapidly evolving pathogens usually differ between the subtrees of its inferred phylogeny, and therefore, relaxed branch-rate models account for the variation in the rate of molecular evolution from clade to clade across the branches of the tree (79). Substitution rates across branches are assumed to be either autocorrelated (80) (i.e., substitution rates are dependent) or uncorrelated (81) (i.e., substitution rates are independent). The uncorrelated branch-rate prior commonly used for rapidly evolving viruses, in which the branch rates are drawn either from exponential or log-normal parent distribution (81). Another alternative to the strict clock model is local molecular clocks, which can estimate different rates for different predefined branch groups within a tree (82). However, for large datasets, the manual task of assigning branches to different groups is impractical (81), and therefore, Bayesian random local clocks can nest a series of local clocks with each extending over a group of branches within the full phylogeny (83).

#### Node-Age Models

Phylogenetic trees are inferred from individually sampled sequences to estimate the statistical properties of the population where the sequences were collected (84). Kingman's n-coalescent theory (i.e., node-age model) is the first stochastic model framework aimed at estimating the size of the sequences' population (85). The theory describes the distribution of coalescent times in the phylogeny as a function of the size of the population from which the sequences were drawn (85). Hence, in the past few decades, the coalescent theory is the core of phylodynamic methods and has shown to be the most useful for inferring essential parameters that shapes the evolution and population dynamics of evolving populations including their effective size (86), rate of growth (87), structure (88), recombination, and reticulate ancestry (89). Expanding the temporal frame of sampling times is the ultimate approach for increasing the statistical power and precision of the coalescent model in estimating substitution rates and population demographics of rapidly evolving viruses (90). An essential evolutionary parameter estimated from the coalescent model is effective population size (*N*_*e*_) at a specific time (*t*) and interpreted as the natural population that represents sample genealogies that have statistical features of an idealized population size through time *N*_*e*_(*t*) (84). However, such interpretation is only suitable for a non-recombinant single population, whereas complex populations with more frequent recombination events require the use of structured tree models (84) described in the following section.

Estimating the posterior phylogeny of a well-mixed population with changing population size can be attained using either parametric or non-parametric node-age models (84). Parametric node-age models accommodate standard continuous population functions, the simplest and most naïve, namely, the constant population growth (CP), which assumes that the population growth rate is zero (91). The other three parametric models include the logistic (LG) growth (assumes the population growth rate is decreasing over time), exponential (EX) growth (assumes the population growth rate is fixed over time), and the expansion (EGx) growth (assumes the population growth rate is increasing over time) (91). One would expect, in the event of an epidemic caused by a rapidly evolving virus like influenza and in the absence of new vaccination, the population growth rate of the virus would realistically fit either an exponential or an expansion growth rate model (44, 92).

Unlike parametric node-age models, non-parametric models can be used to visually infer the history of population size through time (i.e., genetic diversity) from the sequence data in terms of inclines and declines (93). These models treat each coalescent interval as a separate segment to represent a parameter for population size in a given time, in which the number of segments can be specified by the investigator to generate a sky plot (93). The piece-wise constant Bayesian skyline (BS) is the simplest non-parametric model, which assumes that the effective population size is experiencing an episodic stepwise changes through time (93). However, the BS model is shown to be very sensitive to the total number of change points (i.e., coalescent intervals) when specified as a prior as well as to the number of sequences sampled at each point of time (94). Hence, a Gaussian Markov random fields Bayesian Skyride (GMRF) was proposed as an alternative model to BS (95). The GMRF model is less sensitive to the prior number of change points because it implements a temporal smoothing approach to recover accurate population size trajectories (95). However, an improved version of the GMRF is the Skygrid (SG), which takes into account mutation parameters of multi-locus sequences (33). The SG provides a more realistic estimate of demographic history in terms of population size and divergence times, as well as flexibility in terms of the ability to specify cut-points to the time trajectories (33). Furthermore, the SG model is the least sensitive to the temporal distribution of sequences (33). A notable example of sky plots utility in PRRS virus molecular surveillance in the United States was demonstrated by Alkhamis et al. (21) and Alkhamis et al. (37). Their sky plot inferred a distinctly high genetic diversity through time for the emerging 1-7-4 RFLP-type PRRV virus (37), while inferred consistent seasonal increases and decreases in the relative genetic diversity through time for endemic strains isolated between 2014 and 2015 (21).

#### Phylogeographic and Other Epidemiological Phylodynamic Models

Mugration models are substitution models used to infer the migration processes of evolving organisms (96). The most notable implementation of a migration model was developed by Lemey et al. (97) using a CTMP to infer H5N1 avian influenza virus's global origins and movements between countries. They used countries from which the sequences have been sampled as discrete traits to estimate migration rates between pairs of predefined sets of geographical locations, and therefore, the method is named discrete phylogeography (97). Also, the method is known as discrete trait analysis (DTA) because it has the flexibility to use any other discrete trait such as host or farm characteristics from which the sequences have been isolated to model migration rates between infected hosts and farms (37, 98). Besides, the method can infer ancestral origins (i.e., from the assigned discrete traits) for the internal nodes of the phylogeny through their estimated root state posterior probabilities (RSPP) (97). However, the most notable feature of discrete phylogeographic models is the integration of a Bayesian stochastic search variable selection (BSSVS) procedure to identify significant viral dispersal routes between geographical regions or host species (97). BSSVS can also infer the significance of the directionality in the migration process between pairs of discrete traits through integrated symmetric and asymmetric substitution models. The symmetric (Sym) model assumes that the transition rate from state “A” to “B” is the same as the transition rate from state “B” to “A” (i.e., directional spread between traits is insignificant), while the asymmetric (Asym) model assumes that the transition rate from state “A” to “B” is different from the transition rate from state “B” to “A” (i.e., directional spread between traits is significant) (97). However, the lack of a sufficient number of sequences closer to the root of the phylogeny can impact accurate estimation of ancestral traits (i.e., ancestral geographical location or host) by the DTA method (97). Therefore, DTA robustness can be improved by increasing the geographical density and temporal depth of sampling (96). DTA is also limited by the type and number of variables that can be used to estimate ancestral states. Therefore, the BSSVS framework has been extended to accommodate a transitional rate matrix between discrete traits as a generalized linear model (GLM) (22, 32). The method improves biological plausibility of the inferred RSPP for the ancestral traitsby simultaneously estimating the inclusion probabilities of geographic, demographic, and environmental predictors (22). However, the method is shown to be more sensitive to sampling bias than the standard BSSVS approach (32). Hence, comparative sensitivity analyses to sampling bias between the approaches are recommended to avoid severely biased inferred RSPPs.

In some settings, geographical boundaries cannot be defined by discrete spatial traits such as the distribution of wildlife hosts or disease vectors and, therefore, viral evolution and spread better modeled by continuous spatial diffusion models (96). When precise geographical information is available (i.e., longitude and latitude), continuous phylogeographic can reconstruct the viral spatio-temporal evolutionary history using relaxed random walk models (19). These models can additionally estimate viral dispersal rate in km^2^/year and can distinguish whether the spatial diffusion process was homogenous (e.g., dispersal by air) or heterogeneous (dispersal by movements) (19, 21).

In many instances, sequence samples tend to cluster within a geographical region leading to incomplete mixing and formation of structure in the population. This might bias the posterior inferences that estimated the coalescent phylogeographic models mentioned above. Hence, the recently developed structured coalescent tree models for inferring phylogeography can simultaneously model the migration process between regions while allowing for those regions to have their unique coalescent rates (96, 99). Unlike BEAST 1.X, BEAST 2.X has recently implemented several structured coalescent models for inferring geographic and between-host transmission histories, including Bayesian structured coalescent approximation (BASTA) (26), structured coalescent transmission tree inference (SCOTTI) (100), and marginal approximation of the structured coalescent (MASCOT) (101).

The complexity of infectious disease transmission dynamics pushed the capacity of phylodynamic models beyond demographic and phylogeographic reconstructions into investigating traditional and new epidemiological problems. One notable example was demonstrated by Volz et al. by developing a structured coalescent susceptible-infected-recovered (SIR) model to infer reproductive numbers from viral sequences data (102). Similar, but more complex, implementations of mathematical epidemiology in the phylodynamic models were described elsewhere (103, 104).

#### Setting and Running Phylodynamic Models

Prior phylodynamic models described above can be readily selected and set using a graphical user interface (GUI) implemented within the BEAST software package, namely, the Bayesian Evolutionary Analysis Utility (BEAUti) (24, 105). After selecting and setting the models, the software generates a standard XML format structured text file allowing for flexible modifications for more sophisticated evolutionary models. However, the generated XML files are very complex in their structure, and therefore, manual modifications should be made by relevant experts to avoid the introduction of significant error into the model (105). Additional tutorials on selecting and setting evolutionary models using BEAST 1.X are available elsewhere (106–108).

Phylodynamic model selection is a critical component of the analysis pipeline described in Figure 1, simply because different pathogens or gene segments have different evolutionary processes. Therefore, using a single phylodynamic model with similar priors to infer the evolution of multiple pathogens may be biologically implausible, leading to biased inferences. Exploring the fit of the sequence data to different phylodynamic model combinations, in terms of substitution, branch rate, and node age to infer divergence times, Time to the Most Recent Common Ancestor (TMRCAs), evolutionary rates is the best strategy for ensuring accurate estimation of posterior inferences. For inferring viral demographic history, our suggested pipeline (Figure 1) leads to the generation of eight phylodynamic model combinations for a single gene segment, including the selected substitution model (by PartitionFinder), two branch rate priors (UCED and UCLN), and four node-age priors (Cp, Ex, Exg, and SG). However, when inferring phylogeographic history using DTA, we suggest exploring both Sym and Asym BSSVS models (Figure 1), which will lead to the generation of 16 models. Our rigorous analytical pipeline is indeed timely and computationally demanding, but on the other hand, it will lead to the selection of the most realistic model that fits the sequence data with confidence. However, this suggested pipeline is not a strict set of procedures that will ensure appropriate inferences, and therefore, researchers may explore other model or analytical pipelines relevant to their evolutionary hypotheses. It is worth noting that the computational efficiency has been substantially improved in BEAST version 1.10 and the accompanied software library Broad-platform Evolutionary Analysis General Likelihood Evaluator (BEAGLE; permits flexible parallel computing) when compared to earlier versions. The fit of the sequence data to the most realistic phylodynamic model can be assessed through simultaneous estimating the marginal likelihood (MLL) using the path sampling (PS) (25) and stepping-stone sampling (SS) (109) implemented in BEAUti using the standard settings (i.e., simulating across 100 samples for 1 million cycle from the posterior to the prior with a prior reflection point of Beta [0.3, 1.0]). The joint posterior probability density of the models' parameters is estimated by the MCMC algorithms. Setting the appropriate length of the MCMC chains (i.e., number of cycles) to ensure model convergence is dependent on the number of sequences in the dataset. One recommended approach is to quadratically increase the chain length relative to the number of sequences (e.g., 4 million states per sequence) (110). Finally, creating duplicate runs from each generated model can aid in assessing the performance stability of the MCMC simulations and their MLL estimates.

#### Evaluating Phylodynamic Models

MCMC log-files generated by BEAST can be thoroughly evaluated using a friendly GUI software known as Tracer (111). The software provides a simultaneous platform for summarizing and visualizing posterior estimates. Appropriate model convergence can be evaluated by examining the MCMC mixing (based on acceptance ratios) using trace plots, after discarding the 10% of the sample (the “burn-in”). Besides, assessing the estimates of the effective sample sizes (ESS) for each parameter, in which ESS values >200, indicates good model convergence (111). On some occasions, good model convergence does not ensure consistent parameter estimation due to the use of non-informative priors implemented in BEAUti. Therefore, it is critical to compare posterior parameter estimates (e.g., evolutionary rates, population growth rates, PS, and SS MLL estimates) between independent runs for each model to warrant that each parameter is closely identical to its duplicate run. In case of improper model convergence and inconsistent parameter estimation, it is recommended to either increase the length of the MCMC chain or the use of informative priors from previous MCMC runs for the same gene segment or pathogen.

Model selection is achieved by comparing the Bayes factor (BF) of the resulting MLL estimates (from the PS and SS methods) of their corresponding candidate models (25). Briefly, the BF value of the candidate models is summarized using a matrix and computed using the following equation:

$$\begin{array}{l} BF=2\left(lnp\left(Y|M_{i}\right)-\;lnp\left(Y|M_{j}\right)\right) \end{array}$$

where *Y* is the sequence data, *M*_*i*_ is the candidate model “*i*,” *M*_*j*_ is the competing candidate model “*j*,” and ln*p*(*Y*|*M*) is the MLL estimate by either SS or PS simulators. BF values estimated by the SS method are summarized on the upper off-diagonal of the matrix, while BF values estimated by the PS method are summarized on the lower off-diagonal of the matrix. A model with horizontal (i.e., row side of the maxtrix) BF values greater than other candidate models is selected. Additional applied examples on model selection using BEAST 1.X are available elsewhere (106–108). The ultimate goal of the model selection procedure is to find the best fitting model that generated the data, while combining simplicity with biological realism, to appropriately represent the evolutionary characteristics of the pathogen under study (25, 112).

### Summary and Visualization of Evolutionary Inferences

Inferred relative genetic diversity through time (or other reconstructed demographic trajectories) and its highest posterior density (HPD) interval can be summarized using sky plots (e.g., Skygrid plot) generated by Tracer. Similarly, estimates of divergence time, TMRCAs, and substitution rate/site/year with their HPD intervals can be summarized in Tracer using either box or violin plots (111). Also, Tracer provides a flexible platform for simultaneous comparison of evolutionary estimates inferred by multiple phylodynamic models.

Next, the resulting marginal posterior probability density of the selected model is summarized as a maximum clade credible (MCC) tree using TreeAnotator (24) to generate a tree file. MCC tree (from the tree file) can be then visualized and annotated with either posterior support values or RSSPs of the discrete traits at the internal nodes using FigTree (113). In addition, FigTree provides many customizable tree visualization options as well as it allows the users to upload additional information using a text file to annotate flexibly descriptions on the nodes and branches of the trees.

SpreaD3 is an interactive Java-based parsing and rendering tool that can summarize and visualize phylodynamic reconstructions to infer spatio-temporal and trait evolutionary history (114). Also, SpreaD3 integrates JavaScript D3 libraries to provide a web-based visualization platform for phylogeographic trees and their related inferences by combining information from the MCC tree and GeoJSON-based geographic map files (114). SpreaD3 can generate a time-lapse that superimposes the MCC tree annotated with either discrete or continuous spatial traits on a map, which can be visualized using either GIS-KLM virtual globe software (e.g., Google Earth) or modern web-browsers (e.g., Safari or Chrome). This time-lapse demonstrates the epidemic reconstruction of pathogen evolutionary history through space and time, which can quantify the diffusion processes within and between geographical regions. Furthermore, SpreaD3 can identify and plot well-supported rates between pairs of discrete traits using BFs estimated from the symmetric or the asymmetric BSSVS models. Statistically significant rates with large BF values can be used to demonstrate critical viral dispersal routes between geographical regions or transmission cycles between host species.

## Worked Example: Evolutionary Dynamics of Swine Influenza in the United States Between 2015 and 2018

### Sequence Data

The spillover of H3 SI virus from humans to swine in the early 2010s in the United States resulted in a novel emerging virulent strain, which was antigenically distinct from endemic swine strains, and therefore was named “human-like” H3 virus (115). Swine-related anthropological activities such as pig movement and vaccination are the most likely factors for the continuous emergence of SI novel strains (6). Therefore, integrating phylodynamic methods with influenza surveillance systems may reduce the continuous evolutionary implications of SI viruses on both public and animal health in the United States and worldwide. Here, we chose DTA models for our comparative phylodynamic analyses example, due to their popularity, ease of use, interpretation, and computational efficiency when compared to more complex similar models.

Hence, we retrieved HA and PB2 nucleotide sequences of human-like H3 SI from the Influenza Research Database (116) to explore their evolutionary history using our suggested phylodynamic pipeline, described above (Figure 1). The data comprised 352 sequences with complete date and geographical information for each gene segment and was collected from 17 U.S. states (Arkansas, Illinois, Indiana, Iowa, Kentucky, Maryland, Michigan, Minnesota, Missouri, North Carolina, Ohio, Oklahoma, Oregon, Pennsylvania, South Dakota, West Virginia, Wisconsin) between January 8, 2015 and June 1, 2018. The sequence data were collected from the swine production systems and exhibition swine agricultural state fairs as part of the United States Department of Agriculture (USDA) swine influenza surveillance program (40) and was partially analyzed by Walia et al. using classical phylogenetic methods (6). We aligned the sequences for both gene segments and assessed the topological (in)congruence of their phylogeny by performing an ML analysis for the individual segments using the GTR + Γ substitution model, which entailed 10 through bootstrap searches with 100 ML replicates in each run (Supplementary Figure 1). For the subsequent phylodynamic analyses, we removed recombinant and 100% identical sequences, which reduced the dataset to 142 sequences for each gene segment (Supplementary Table 1). We then evaluated the fit of the sequences to the most realistic substitution model and partitioning scheme using the BIC approach. Finally, we evaluated the temporal signal in the sequence data and found that both segments were suitable for the subsequent molecular clock analyses (*R*^2^ = 0.65 and 0.40 for HA and PB2, respectively) (Supplementary Figure 2).

### Comparative Phylodynamic Analyses

We assessed the sensitivity of the inferred posterior evolutionary of human-like H3 SI sequence data to the choice of different gene segments (i.e., HA vs. PB2) and phylodynamic priors, including substitution, discrete spatial trait, branch rate, and node-age models on the (Figure 1). For each gene segment, we generated 16 phylodynamic models (a total of 32 runs for both segments) using the default none-informative priors' combinations implemented in BEAUTi (Figure 1). These prior models included: (1) the GTR + Γ vs. the HKY + Γ for the site models; (2) the symmetric vs. asymmetric for discrete spatial models; (3) the UCLN vs. UCED for the clock models; and (4) the CP vs. The EG vs. The EGx vs. the SG for the coalescent tree models (Figure 1). We excluded spatial traits (i.e., U.S. states) with only one sequence (Supplementary Table 2) leading to the inclusion of 10 states in the subsequent DTA. Also, we evaluated the fit of the 16 phylodynamic models to the HA and PB2 sequences using the BF comparisons of their MLL estimated by the PS and SS simulator in order to select the most realistic model and correctly interpret its posterior inferences. We then used two replicate MCMC simulations for 150 million cycles and sampled every 1,500th state for each candidate model.

After assessing for proper model convergence, we compared the inferred evolutionary demographics of each candidate model by summarizing their inferred divergence times, substitution rates, and TMRCAs. Besides, we then generated the SG plots to compare relative genetic diversity for HA and PB2 gene segments inferred from the two different sites and discrete spatial models. Similarly, we compared the phylogeographic inferences of each model by generating MCC trees, summarizing the RSPPs of the states, and plotting them at the internal nodes of their corresponding trees. Finally, we selected and plotted the statistically significant dispersal routes between states under each candidate model using a cutoff BSSVS-BF ≥ 10.

### Results

#### Demographic Posterior Inferences of HA and PB2 Gene Segments

**Figure 2:**
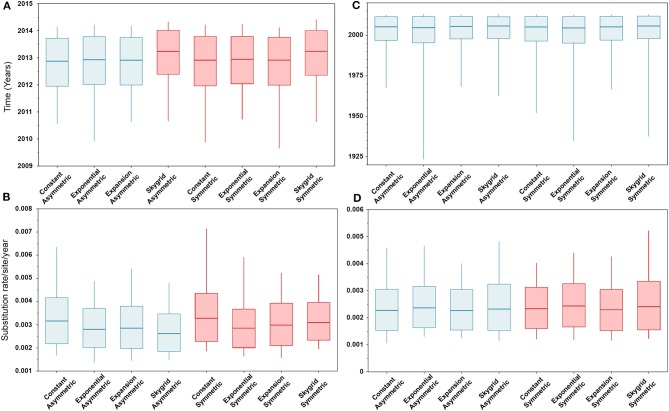
Box plots of divergence times and evolutionary rates (substitution rate/site/year) of hemagglutinin (HA) and polymerase basic 2 (PB2) gene segments of human-like H3 swine influenza virus collected between January 2015 and June 2018 in the United States. The boxplots summarize the posterior estimates of eight phylodynamic model combinations (node-age and BSSVS priors) for each gene segment. Boxes represent 95% high posterior density (HPD), and midlines indicate the posterior median for each estimate. Blue and red boxes indicate symmetric and asymmetric models, respectively. **(A)** Divergence times of HA gene. **(B)** Substitution rates/site/year of HA gene. **(C)** Divergence times of PB2 gene. **(D)** Substitution rates/site/year of PB2 gene.

The BIC values, described above, indicated that the HKY + Γ is the best fitting substitution model for the HA gene segment (BIC = 13,399), while the GTR + Γ is the best fitting substitution model for the PB2 gene segment (BIC = 20,029). In addition, results of the BF values (≥5) indicates that the best fitting branch-rate and node-age models to the sequence data were the SG + UCLN for HA and SG + UCED for PB2 segments (Supplementary Tables 3–6). However, there were no significant changes in the posterior demographic inferences when choosing the opposite substitution model for both gene segments. Similarly, our results indicate that the choice of discrete spatial and node-age models does not substantially change the estimated divergence times and substitution rates/site/year (Figure 2) for each gene segment alone. Additionally, these estimates were also not sensitive to the choice of branch-rate models (i.e., UCED and UCLN). However, when comparing divergence times between segments, our results indicate substantial differences in a magnitude of ~8 years, in which the divergence time for the HA segment was around 2013 (Figure 2A), while for the PB2 segment, it was around 2005 (Figure 2C). No differences were observed in the substitution rates/site/year between the two gene segments, which were ranging between 3.3 × 10^−3^ (95% HPD; from 2.8 × 10^−3^ to 3.9 × 10^−3^) and 2.9 × 10^−3^ (95% HPD; from 2.2 × 10^−3^ to 3.8 × 10^−3^) for HA and PB2 segments, respectively (Figures 2B,D).

**Figure 3:**
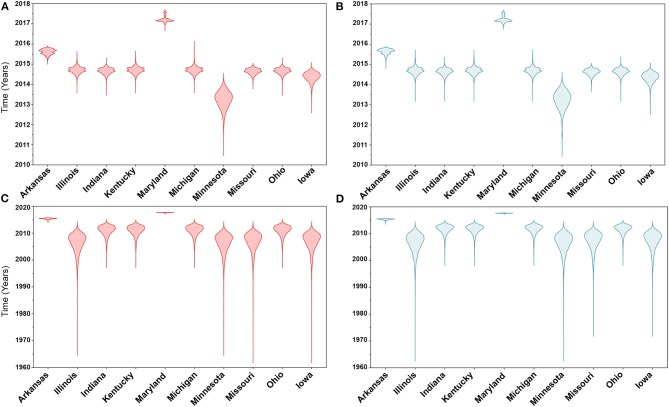
Violin plots of state level time to the most recent common ancestors (TMRCA) of hemagglutinin (HA) and polymerase basic 2 (PB2) gene segments of human-like H3 swine influenza virus collected between January 2015 and June 2018 in the United States. The plots were generated from the Skygrid coalescent tree model. Red and blue colors indicate the asymmetric and symmetric BSSVS priors, respectively. **(A)** TMRCAs of HA gene estimated from the asymmetric BSSVS model; **(B)** TMRCAs of HA gene estimated from the symmetric BSSVS model; **(C)** TMRCAs of PB2 gene estimated from the asymmetric BSSVS model; **(D)** TMRCAs of PB2 gene estimated from the symmetric BSSVS model.

**Figure 4:**
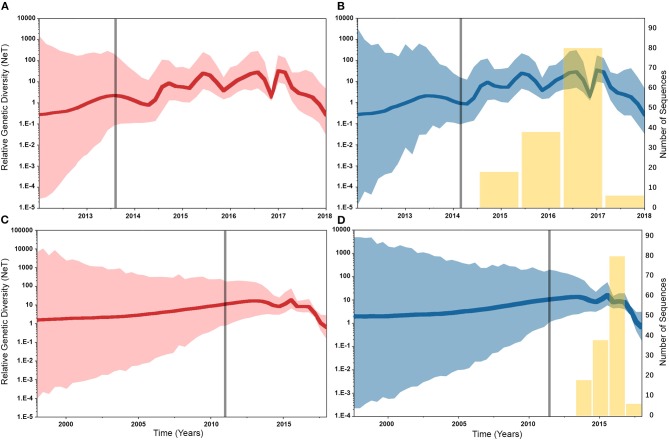
Bayesian Skygrid (SG) plots of the relative genetic diversity through time of hemagglutinin (HA) and polymerase basic 2 (PB2) segments of human-like H3 swine influenza virus collected between January 2015 and June 2018 in the United States. The dark line indicates the posterior median estimate, and the 95% high posterior density (HPD) is indicated by the light shaded areas (red and blue for the asymmetric and symmetric BSSVS priors, respectively). The vertical gray line indicates the estimated time at which the relative genetic diversity transitioned from a slow to a fast growth rate. Yellow bars indicate the temporal distribution of the sequence data. **(A)** SG plot of HA gene inferred from the asymmetric BSSVS prior. **(B)** SG plot of HA gene inferred from the symmetric BSSVS prior. **(C)** SG plot of PB2 gene inferred from the asymmetric BSSVS prior; **(D)** SG plot of PB2 gene inferred from the symmetric BSSVS prior.

Similarly, posterior estimates of TMRCAs were not sensitive to the choice of phylodynamic priors but were different between the two gene segments (Figure 3). Hence, based on the HA segment, our results hint that the oldest human-like H3 strains emerged from the state of Minnesota in mid-2013 (Figures 3A,B), but with a notable overlap in the 95% HPD of the TMRCAs inferred for other states (excluding Maryland). However, results distinctly suggest that the youngest strains emerged from the state of Maryland in early 2017. Results of the PB2 segment were inconclusive in terms of determining the oldest strains, but identical to the HA gene in identifying Maryland as the state of the youngest viral strains (Figures 3C,D). Also, the choice of spatial trait model did not affect our estimates of genetic diversity for both HA and PB2 segments (Figure 4). Our SG plots inferred seasonal variations in terms of increases and decreases, in the genetic diversity through time for HA segments (Figures 4A,B), while the genetic diversity of the PB2 segment gene slightly declined after 2015 (Figures 4C,D).

#### Phylogeographic Posterior Inferences of HA and PB2 Gene Segments

**Figure 5:**
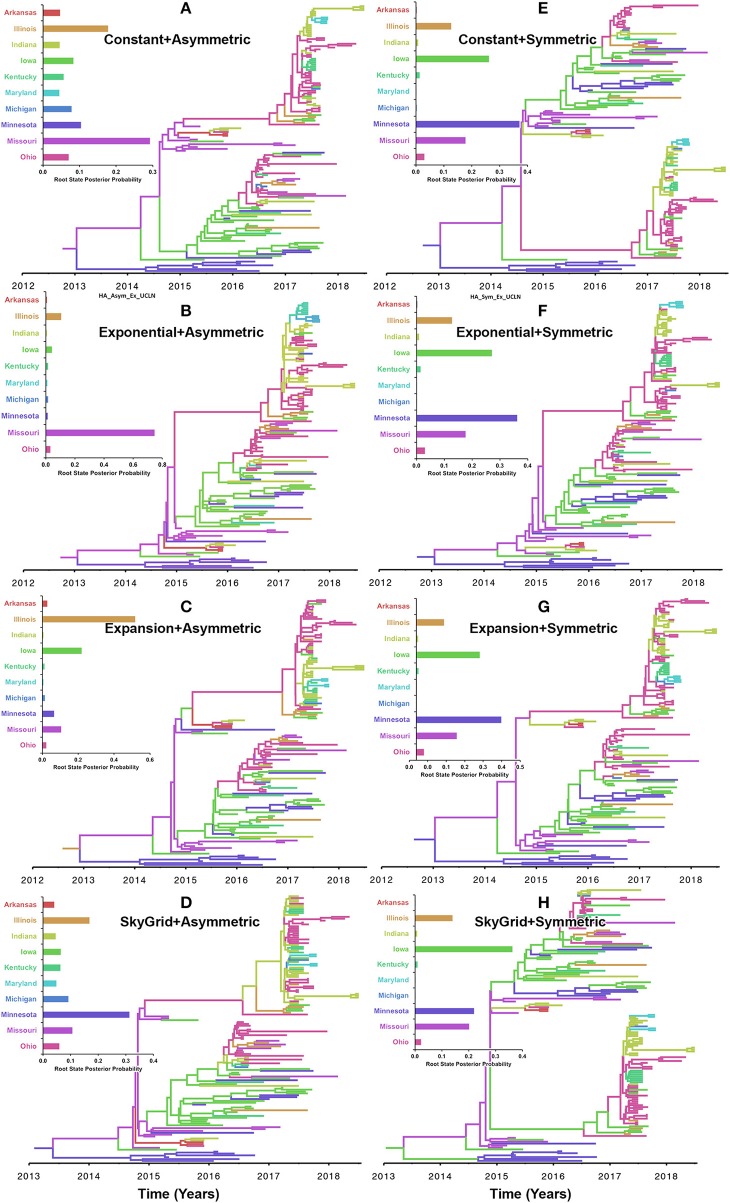
Maximum clade credibility (MCC) phylogeny of the HA segment of human-like H3 swine influenza virus collected between January 2015 and June 2018 in the United States. The trees are inferred from eight phylodynamic model combinations (node-age and BSSVS priors). The color of the branches represents the most probable location state of their descendant nodes, and their color-coding corresponds to the upper left bar chart, which represents the root location state posterior probabilities (RSPP) for each state. **(A–D)** Trees inferred from four node-age + asymmetric BSSVS priors. **(E–H)** Trees inferred from four node-age + symmetric BSSVS priors.

Our inferred phylogeographic posteriors did not show sensitivity to the selection of substitution or molecular clock priors. However, substantial differences were inferred when selecting different node-age and discrete spatial trait priors. Inferences from both the CP and the EX node age with the asymmetric models implicated Missouri as the most likely ancestral state for the human-like H3 virus currently circulating in the United States when using the HA gene segment (Figures 5A,B). However, the EGx and the SG with the asymmetric models Illinois and Minnesota as the most likely ancestral states, respectively (Figures 5C,D). Yet, when using the HA segment, the symmetric model with the CP, EG, and EGx priors consistently implicated Minnesota with approximately similar estimates of RSPPs (Figures 5E–G). In contrast, the use of the symmetric model with the SG prior implicated Iowa as the ancestral location for the currently circulating human-like H3 strains (RSPP = 0.36) (Figure 5H). Interestingly, the HA sequence data uniquely favored this prior combination when using the BF comparisons for the best fitting phylodynamic model (Supplementary Tables 3, 4).

**Figure 6:**
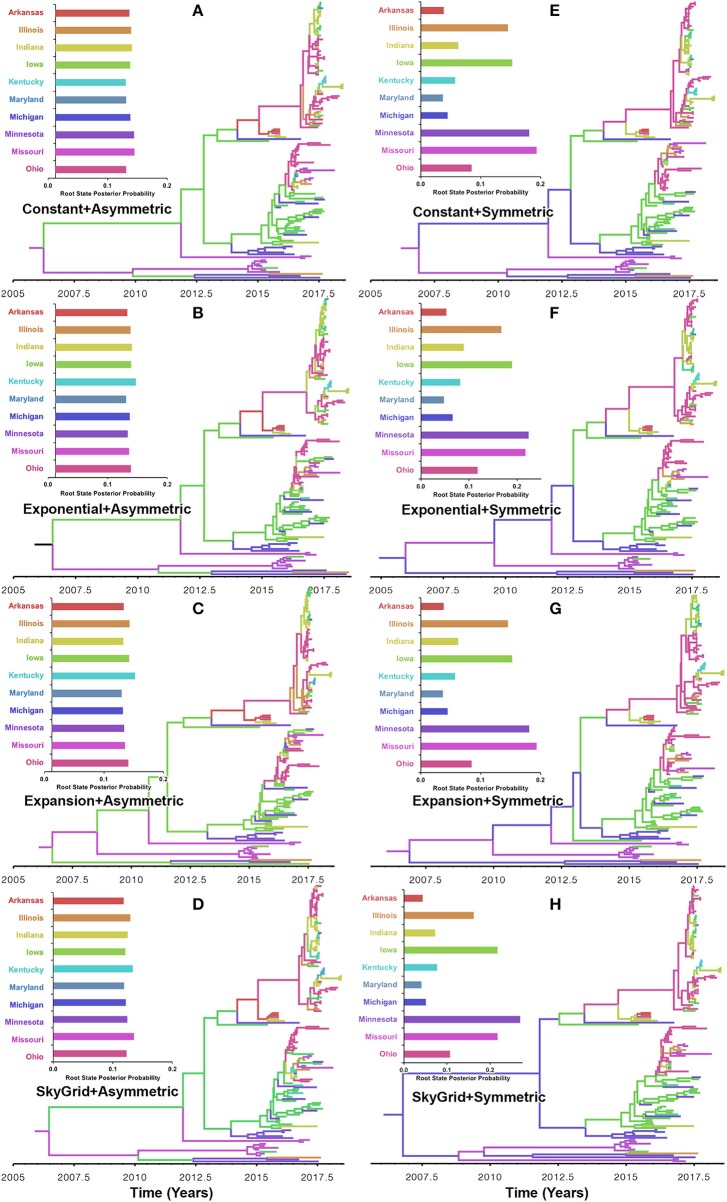
Maximum clade credibility (MCC) phylogeny of the PB2 segment of human-like H3 swine influenza virus collected between January 2015 and June 2018 in the United States. The trees are inferred from eight phylodynamic model combinations (node-age and BSSVS priors). The color of the branches represents the most probable location state of their descendant nodes, and their color-coding corresponds to the upper left bar chart, which represents the root location state posterior probabilities (RSPP) for each state. **(A–D)** Trees inferred from four node-age + asymmetric BSSVS priors. **(E–H)** Trees inferred from four node-age + symmetric BSSVS priors.

Our BF values suggested that the PB2 sequence data favored the asymmetric model with the SG prior, but with a very slight edge over the symmetric model with the same coalescent prior (Supplementary Tables 5, 6). RSPPs inferred from the PB2 segment were almost equal for all states and, hence, were inconclusive, when using the asymmetric model with the four coalescent priors (Figures 6A–D). Similarly, using the symmetric model with the four coalescent priors was inconclusive in terms of identifying the ancestral location for the currently circulating viral strains (Figures 6E–H). More specifically, the magnitude of differences between Minnesota and Missouri and in the inferred RSPPs, across different coalescent priors, was substantially small (Figures 6E–H). For example, when using the SG prior, the inferred RSPPs were 0.18 and 0.22 for Missouri and Minnesota, respectively (Figure 6H).

**Figure 7:**
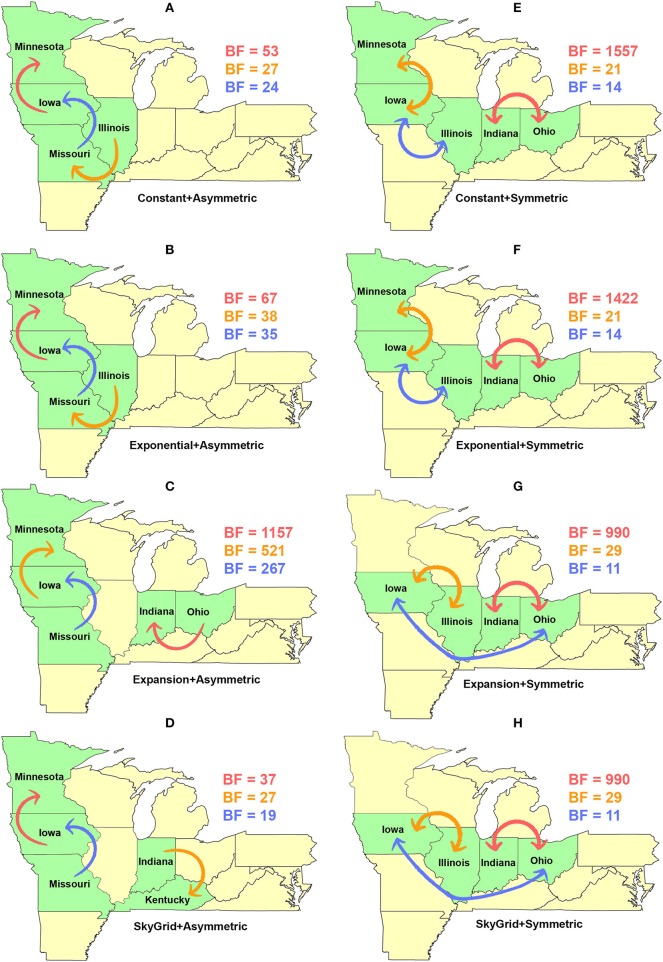
Dispersal routes of human-like H3 swine influenza virus between states inferred from the HA gene segment. Dispersal routes with non-zero rates were inferred using the Bayesian stochastic search variable selection (BSSVS) approach, and statistically significant routes were selected using Bayes factors (BF). The top three dispersal routes with the strongest statistical support (by the BFs) are plotted. Arrows' colors correspond to the color legend of their BF values on the upper right of each map. **(A–D)** Dispersal routes inferred from four node-age + asymmetric BSSVS priors. **(E–H)** Dispersal routes inferred from four node-age + symmetric BSSVS priors.

Our BF-BSSVS analyses, using the asymmetric model with the CP and the EX coalescent priors for the HA gene segment, suggest that the top three most significant unidirectional routes of viral dispersal (BF > 18) were between Minnesota, Iowa, Illinois, and Missouri (Figures 7A,B). The inferred routes maintained their unidirectionality from the origin to the destination geographical locations, using CP and EX priors (Figures 7A,B). Similarly, the order of statistical significance suggests that the route from Iowa to Minnesota is the most important for viral dispersal between states (Figures 7A,B). In contrast, the EXg with the asymmetric model suggests that the route from Ohia to Indiana is substantially the most significant dispersal route (BSSVS-BF = 1,157) (Figure 7C). Nevertheless, the SG prior agrees with the results of the CP and EX priors in inferring the route from Iowa to Minnesota as the most significant (BSSVS-BF = 37) (Figure 7D), while inferences from the symmetric model and the four coalescent priors consistently agreed that the top most significant bidirectional route of viral dispersal (BF ≥ 990) was between Indiana and Ohio (Figures 7E–H). However, disagreements were inferred on the second and the third most significant routes when using the CP and EX on one side and EXg and SG on the other (Figure 7H).

**Figure 8:**
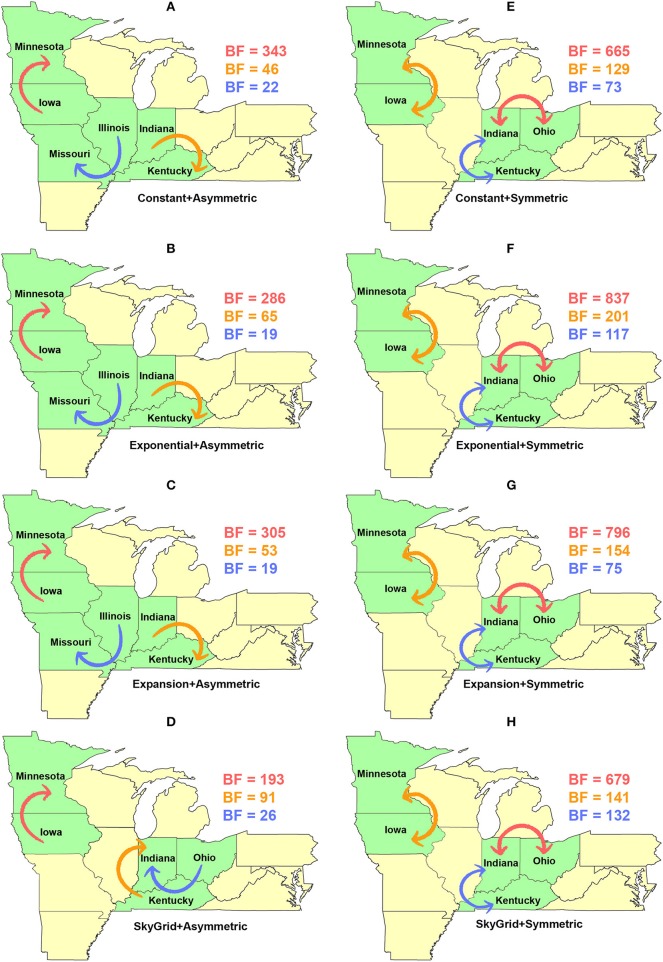
Dispersal routes of human-like H3 swine influenza virus between states inferred from the PB2 gene segment. Dispersal routes with non-zero rates were inferred using the Bayesian stochastic search variable selection (BSSVS) approach, and statistically significant routes were selected using Bayes factors (BF). The top three dispersal routes with the strongest statistical support (by the BFs) are plotted. Arrows' colors correspond to the color legend of their BF values on the upper right of each map. **(A–D)** Dispersal routes inferred from four node-age + asymmetric BSSVS priors. **(E–H)** Dispersal routes inferred from four node-age + symmetric BSSVS priors.

Dispersal routes inferred for PB2 (including the order of significance) were also sensitive to the selected discrete spatial model and slightly to the coalescent priors (Figure 8). Thus, when using the asymmetric model, the top two unidirectional routes included (1) Iowa → Minnesota; (2) Indiana → Kentucky (Figures 8A–D). While the CP, EX, and EXg inferred the route from Illinois to Missouri as the third most significant route (Figures 8A–C), the SG prior inferred the route from Ohio to Indiana as the third most significant route (Figure 8D). Finally, our inferred top three significant dispersal routes were from the symmetric model between (1) Indiana and Ohio; (2) Minnesota and Iowa; (3) Indiana and Kentucky (Figures 8E–H).

## Discussion

In the past decade, our phylodynamic pipeline became well-established and demonstrated powerful potentials to trace the evolutionary history of both animal and human pathogens making it an ideal tool for designing new molecular surveillance systems. In this study, we revisited essential concepts and definitions within the field of phylodynamic methods. Also, we challenged the robustness of the posterior evolutionary parameters, inferred by the commonly used phylodynamic models, using two gene segments, of the currently circulating human-like H3 SI viruses isolated in the United States, and multiple priors. Subsequently, we compared similarities and differences between the posterior parameters inferred from HA and PB2 sequence data using multiple phylodynamic models. Hence, we explored the robust and sensitive aspects of SI phylodynamic models and highlighted the importance of model selection within their analytical framework. However, unlike classical phylogenetic methods currently implemented within the SI surveillance system in the United States, we were able to reveal higher resolution insights into the evolutionary epidemiology of human-like H3 viruses by quantifying their demographic and phylogeographic history. Therefore, animal health researchers and stakeholders need to be aware of the method's features, strengths, and limitations for generating reliable inference to guide future disease intervention activities properly.

### Updated Insights in the Evolutionary Epidemiology of Swine Influenza in the U.S.

Based on the results of the best fitting phylodynamic models for both HA and PB2 segments, evolutionary rates of currently circulating human-like H3 viruses in the United States remain high with no apparent signs of substantial declines (Figures 2B,D) and were similar to what was inferred elsewhere (117). Furthermore, inferred relative genetic diversity through time did not decline for the HA segment and showed evidence of seasonal variation between 2014 and 2018 (Figures 4A,B), while a slight decline in the genetic diversity was inferred for the PB2 segment between 2015 and 2018 (Figures 4C,D). These findings suggest that currently circulating human-like H3 viruses will continue evolutionary activity leading to the generation of novel strains, which is attributed to the frequent and continuous exchange of viruses between commercial and exhibition swine operations in the United States with the later as the epicenter of that exchange (117). Our estimates of the TMRCAs for HA segment slightly agree on the notion that the oldest H3 viruses diverged from earlier outbreaks in the state of Minnesota, which is a central region for the swine industry in the United States (Figure 3). However, the notable overlap in the inferred 95% HPDs of the TMRCAs between most states (Figure 3B) suggest that the currently circulating strains are shifting their evolutionary dynamics in terms of re-emergence and dispersal when compared to earlier strains. Additionally, both gene segments agree on the assumption that H3 outbreaks were recently introduced into the state of Maryland (Figure 3).

The state of Minnesota was inferred to be the ancestral location of human-like H3 viruses isolated from outbreaks observed between 2009 and 2012 (118), which agrees with our TMRCAs inferred from HA segment (Figure 3). However, results of the SG + UCLN symmetric model, selected as the best fitting model for HA sequence data (Supplementary Table 4), implicates the state of Iowa as the ancestral region (after 2013) for currently circulating human-like H3 viruses, followed by the state of Minnesota as a secondary ancestral location (Figure 5H). This is not surprising since Iowa and Minnesota share the most prominent swine production system in the United States with the highest swine density, unrestricted and intense movement of animals between states. Although Iowa and Minnesota are the original hotspots of H3 viruses, our BSSVS BF results showed a markedly significant viral dispersal route between Indiana and Ohio (BF = 990) (Figure 7H). This suggests that the H3 viral gene flow between Ohio and Indiana, inferred for 2009–2012 viruses remains a vital migration route since, particularly within exhibition swine populations (117). Even though Illinois and Indiana formulate one swine production system, there was no significant viral dispersal route inferred between the states. Despite the continuous nature of animal movement within the production system of Minnesota and Iowa, no significant dispersal route was inferred between the two states using the HA segment (Figure 7H). Nevertheless, using the PB2 segment, a highly significant dispersal route was inferred from Iowa to Minnesota, suggesting that Iowa might be the new epicenter for virus dispersal of the currently circulating H3 lineages (Figure 8D). This result is further supported by the significant migration route between Iowa on one side and Illinois and Ohio on the other when using the HA segment (Figure 7H). Also, the inferred dispersal route between Iowa and Illinois (Figure 7H) may reflect interstate movements of exhibition pigs (119). Hence, the movements of exhibition pigs across the United States possibly led to expanding the spatial spread of H3 viruses to states with limited swine production systems (117).

Unlike the HA segment, RSPPs inferred from the most realistic phylodynamic model for PB2 sequences (i.e., asymmetric + SG + UCED) (Supplementary Table 5) did not yield conclusive results about the ancestral geographical origin of human-like H3 in the United States (Figure 6D). Instead, this result demonstrates a homogenous spatio-temporal diffusion process of the PB2 gene between states (Figure 6D), suggesting that the virus has maintained an endemic status across the United States after 2010. Also, results of the SG plot for PB2, described above, showed an overall stationarity in its genetic diversity through time (despite the slight early incline and later decline) (Figure 4C), when compared to the HA gene (Figures 4A,B), supporting the notion of endemic status. However, using the PB2 segment, we inferred a notably significant dispersal route originating from Iowa to Minnesota (BSSVS = 193) (Figure 8D), reflecting a well-established swine transportation route within a production system, as described above. However, this route was not inferred as significant when using the best fitting model for the HA segment (Figure 7H). These results may be attributed to the fact that PB2 evolutionary dynamics are moderately slower than the HA segment (Figure 2) in terms of strength of the temporal signal (Supplementary Figure 2), substitution rate (Figure 2), and age of the segment (Figure 3). Therefore, the PB2 segment maintained similar evolutionary dynamics to earlier strains that emerged in Minnesota and dispersed into Iowa (120). Yet, both HA and PB2 segment agree on the importance of Iowa as a geographical region for dispersal of currently circulating H3 lineages (Figures 7H, 8D). Additionally, we inferred two significant viral dispersal routes originating from Kentucky to Indiana and from Ohio to Indiana (Figure 8D), which further supports the role of exhibition of swine movements between states in maintaining the spread of H3 viruses. Both dispersal routes are mainly maintained by the annual agricultural fairs where exhibition susceptible swine and humans from these states are frequently exposed to direct and indirect contacts from the same infected hosts (121). It is worth noting that the route from Kentucky to Indiana was hypothesized to be important for H3 gene flow between states, but past evolutionary analyses did not observe it due to the lack of sufficient samples (117).

### Robustness and Limitations of Phylodynamic Methods

The uneven sampling of sequences in terms of temporal depth and frequency of associated discrete traits is an inherent limitation of most phylodynamic studies. For example, the inclusion of many recent sequences from a single geographical location may lead to a biased bottleneck effect in the shape of inferred population size through time when using a coalescent model from the Skyline family (122). This issue can be resolved by designing studies with uniform probability sampling with respect to space and time (122). Further, setting DTA is user friendly and computationally more efficient when compared to more complex coalescent models, but it underlays a few assumptions, such as that the sequence sample size is proportional to the size of the selected discrete state (26). Thus, including sequences from severely undersampled discrete traits will tend to produce unreliable posterior inferences, where for example, inferred RSPPs will be skewed toward oversampled areas. Nevertheless, undersampling is a common problem, especially in passive surveillance data, and therefore, the use of structural coalescent models (e.g., BASTA) might be more appropriate (26).

Despite this inherited sensitivity of phylodynamic methods to uneven sampling, our posterior inference from the best fitting models showed remarkable robustness toward such limitation. Although the largest number of collected sequences was in 2017 (80) (Supplementary Table 2), estimates of relative genetic diversity through time did not show any striking jumps in that year for both HA and PB2 segments (Figures 4B, 2D). Additionally, for the HA gene, Iowa (with 26 sequences) rather than Ohio (39 sequences) was inferred as the ancestral location (Figure 5H, Supplementary Table 2). However, seven out of the 17 U.S. states were excluded from the DTA due to the lack of sufficient sequences, and therefore, their role was unquantified in shaping the spatio-temporal evolution of SI. Yet, these states had substantially fewer swine-related activities as well as SI outbreaks than analyzed states.

Further, we showed how the posterior estimates of demographic reconstruction were almost insensitive to the choice of different phylodynamic priors for each gene segment (Figures 2–4). However, inferred evolutionary estimates from different gene regions may differ (41) or coincide (118) due to the natural variation in their mutation rate over time. This raises the question of whether using longer gene segments or whole genomes provides deeper resolution into the evolutionary history of rapidly evolving pathogens. Past influenza A studies (41, 123, 124), including the present study, showed that HA and NA segments typically exhibit higher evolutionary rates than more conserved segments like PB1 and PB2. Subsequently, segments with higher evolutionary rate will also display stronger evolutionary signals, as described above. In our analyses, the width of the 95% HPDs (i.e., length of the time scale) for the median age and TMRCAs of PB2 were remarkably wider than the HA segment (Figures 2, 3). This sizeable width of the posterior intervals reflects the magnitude of uncertainty surrounding inferences from the PB2 segment, as well as suggests that inferences from the HA segment were more precise (or robust) than the PB2 segment. Also, we demonstrated how the PB2 segment failed to identify the ancestral geographical location of currently circulating H3 viruses (Figure 6D). While, using the symmetric model, we inferred four candidate ancestral locations with inconclusive RSPPs (Figure 6H). Further, Nelson et al. (117) were not able to infer a significant migration route between Indiana and Ohio using the PB2 segment. Yet, we were able to infer this particular route as significant using both the HA and the PB2 segments (Figures 7H, 8D). Additionally, Scotch et al. (118) confirmed agreements in the phylogeographic inferences between HA and NA gene segments. This highlights another decisive question about the suitability and efficiency of using single, multiple, or whole genome when using phylodynamic methods for molecular surveillance of viral diseases. Most researchers advocate for whole-genome analysis by either analyzing each segment alone or as concinnated segments. However, in the presence of a large number of sequences, these strategies are ill timed and require massive computational resources, making them inefficient for targeted and near-real-time surveillance systems. It is worth noting that substitution rate and divergence time inferred by Alkhamis et al. (43) using the FMD SAT1 VP1 segment were similar to the evolutionary estimates inferred by Lasecka-Dykes et al. (125) using whole-genome sequences, confirming the robustness of phylodynamic methods. Nevertheless, the presence of recombination events can severely impact the robustness of phylodynamic methods leading to inferring biased evolutionary histories (126). Hence, targeting the most rapidly evolving gene segment at the beginning of an epidemic may suffice molecular surveillance activities. That said, the choice between gene segments or the whole genome should depend on the evolutionary properties of the pathogen, frequency of recombination events, availability of resources, and objectives of the molecular surveillance system.

As described above, phylodynamic inferences tend to be biased toward the available subsets of sequences data. Hence, when analyzing novel sequence datasets, it is critical to combine them with genetically related lineages published in the scientific literature or publicly available databases to reduce the impact of sampling bias as well as improve the reliability and accuracy of posterior evolutionary inferences. Unfortunately, several examples published in the scientific literature used phylodynamic methods on novel sequence datasets while ignoring their published relatives (127–129). This led to inferring MCC trees with unaccounted phylogenetic relationships such as nodes, branches, and roots.

Our worked example opens considerations for future work involving the use of more complex phylodynamic models, described above, to shed deeper insights into the evolutionary epidemiology of SI. For example, when the exact geographical locations of the sequences are available, the use of continuous phylogeographic models will enable us to include all states in the analyses, including states with few sequences. Besides, we can estimate the spatiotemporal dispersal speed of the virus as well as identify dispersal patterns (i.e., homogeneous vs. heterogeneous) across different geographical regions. Also, the use of GLM geographical models can directly quantify the importance of different environmental (e.g., climate) and demographical (e.g., pig density) factors in shaping the evolutionary history of SI in the United States. Finally, exploring the potentials of structured coalescent models in improving the reliability of inferences derived from basic DTAs should be considered as well.

### Future of Phylodynamic Methods for Molecular Surveillance of Animal Diseases

The current surveillance programs rely heavily on collecting and analyzing spatial, temporal, and genomic aspects of an outbreak using classical statistical methods in a disjointed analytical framework. This disjointed framework suffers from many biases and is not capable of answering more profound epidemiological questions about the outbreak of current dynamics. Using our suggested phylodynamic analytical pipeline, we were able to fulfill critical epidemiological questions about the emergence and evolution of currently circulating human-like H3 SI viruses in the United States, with the primary goal of guiding risk-based surveillance resources. For example, using inferences from the HA segment, we were able to identify the dates of epidemic introduction to each state. Also, we were able to identify the geographic origins of the current outbreaks and observed their genomic-spatio-temporal diffusion process through time between states. Also, we identified high-risk viral dispersal routes between states, rank-ordered their significance, and defined their directions. All of these are integral components of an effective risk-based molecular surveillance program, and the ability to achieve in real time is the future molecular surveillance of animal diseases. Nevertheless, the availability of computational resources for designing an ongoing phylodynamic-based molecular surveillance system will always remain a challenge, especially for developing countries. That said, a few open-source software developed recently can perform basic phylodynamic analysis (e.g., estimate molecular clocks and infer evolutionary models) using an ML statistical framework, including TimeTree (130), treedater R package (131), and Least Square Dating (120). While the algorithms implemented in these software trades off the advantages of the Bayesian framework, in the presence of large sequence datasets, they can produce evolutionary estimates similar to those estimated by BEAST using substantially less computational resources (120, 130, 131).

Nextstrain (https://nextstrain.org), which implements TreeTime is a futuristic working example of a web-based real-time molecular surveillance system for important human pathogens such as influenza, Ebola, Dengue, and the newly emerging corona (COVID-19) viruses. This surveillance system has an on-going phylodynamic analytical engine that traces, in real-time genetic diversity, divergence times, geographical origins, and dispersal on global scales. The system updates the results of the MCC tree once new sequences are deposited in other web-based publicly available genomic databases. However, this project is achieved through rigorous and consistent global collaboration and data sharing. In the United States, resources for developing a similar system for tracing animal diseases are readily available. Nevertheless, the chain of collaboration between researchers, government, and producers in the animal sector is hard to maintain due to logistic, economic, and educational (i.e., lack of awareness and skill in phylodynamic methods) reasons. Nevertheless, recent scientific literature on the use of phylodynamic methods for animal disease surveillance is notably growing, which reflects the increased awareness between veterinarians about the capacities of such methods and the goodwill of the industry leaders to voluntarily share their data (37, 132). Therefore, we anticipate a new era of animal disease prevention and control in the United States. In contrast, veterinary infrastructure in developing countries is severely lacking, in terms of reporting and data sharing, when compared to their human health sectors. Consequently, the question related to the future of implementing phylodynamic methods in global animal surveillance remains unanswered.

## Conclusions

Our selected phylodynamic analytical pipeline offers an integrated approach to not only answering more profound epidemiological questions about emerging and endemic animal diseases but also attempts to reduce the impact of its inherent limitations to offer less biased and biologically plausible inferences about the pathogen evolutionary characteristics to properly guide intervention activities. This study has highlighted the value of phylodynamic methods in improving current and future molecular surveillance efforts against animal diseases using human-like H3 SI virus as a working example. We reviewed and outlined basic concepts and aspects of phylodynamic methods and attempted to summarize essential components of the methodology in one analytical pipeline to facilitate the proper use of the methods by animal health researchers. We also pinpointed requirements and challenges for integrating phylodynamic methods in routine animal disease surveillance activities.

## Data Availability Statement

The datasets analyzed for this study (Alignments, BEAST xmls, and MCC tree files) can be found in the Figshare Dataset. https://doi.org/10.6084/m9.figshare.11842989.v1.

## Author Contributions

The study was designed by MA and MT. The data were collected and organized by CL. All statistical analyses were conducted by MA. MA, CL, and MT wrote the first draft of the manuscript.

## Conflict of Interest

The authors declare that the research was conducted in the absence of any commercial or financial relationships that could be construed as a potential conflict of interest.
