# Supplementary material for: Animal Disease Surveillance in the 21st Century: Applications and Robustness of Phylodynamic Methods in Recent U.S. Human-Like H3 Swine Influenza Outbreaks
Source: Front Vet Sci. 2020 Apr 21;7:176. doi: 10.3389/fvets.2020.00176 (PMC7186338; doi:10.3389/fvets.2020.00176)
Supplement: Supplementary file 1 [file Data_Sheet_1.docx]

Supplementary Material

Exploring the Features and Prospects of Phylodynamic Methods for Animal Disease Surveillance: Swine Influenza in United States as a Case Study

Moh A. Alkhamis ^1,2*^, Chong Li^2^, Montserrat Torremorell^2^

# Supplementary Data

Supplementary Material should be uploaded separately on submission. Please include any supplementary data, figures and/or tables. All supplementary files are deposited to FigShare for permanent storage and receive a DOI.

# Supplementary Figures and Tables

## Supplementary Figures


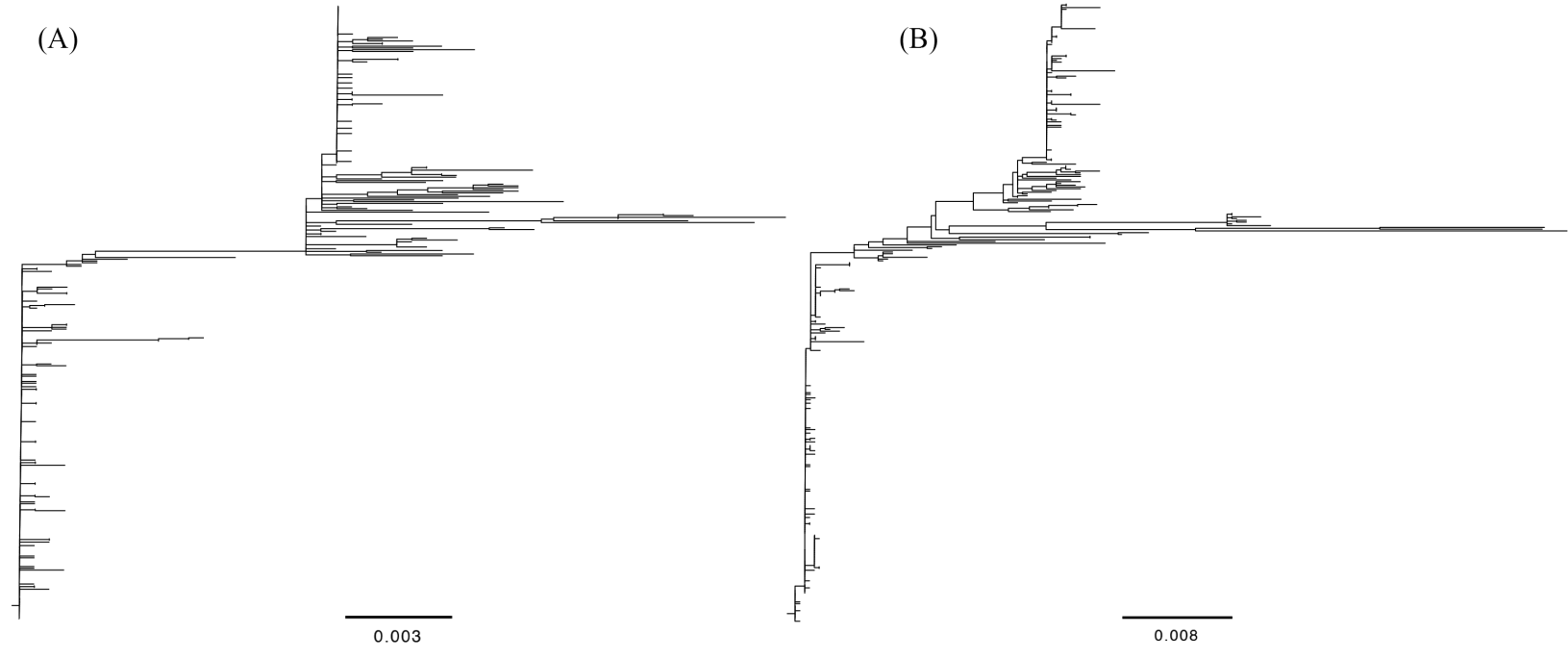


**Supplementary Figure 1.** **Maximum likelihood (ML) phylogeny of HA and PB2 gene segments of human-like swine influenza virus between January 2015 and June 2018 in the USA.** The trees were estimated using the GTR+Γ substitution model implemented in RAxML version 8. Support given at nodes were based on 10 through bootstrap search with 100 ML replicates in each run. Scale bar indicate substitution rate per site. (A) represents HA ML tree, (B) represents PB2 tree. (N = 352).


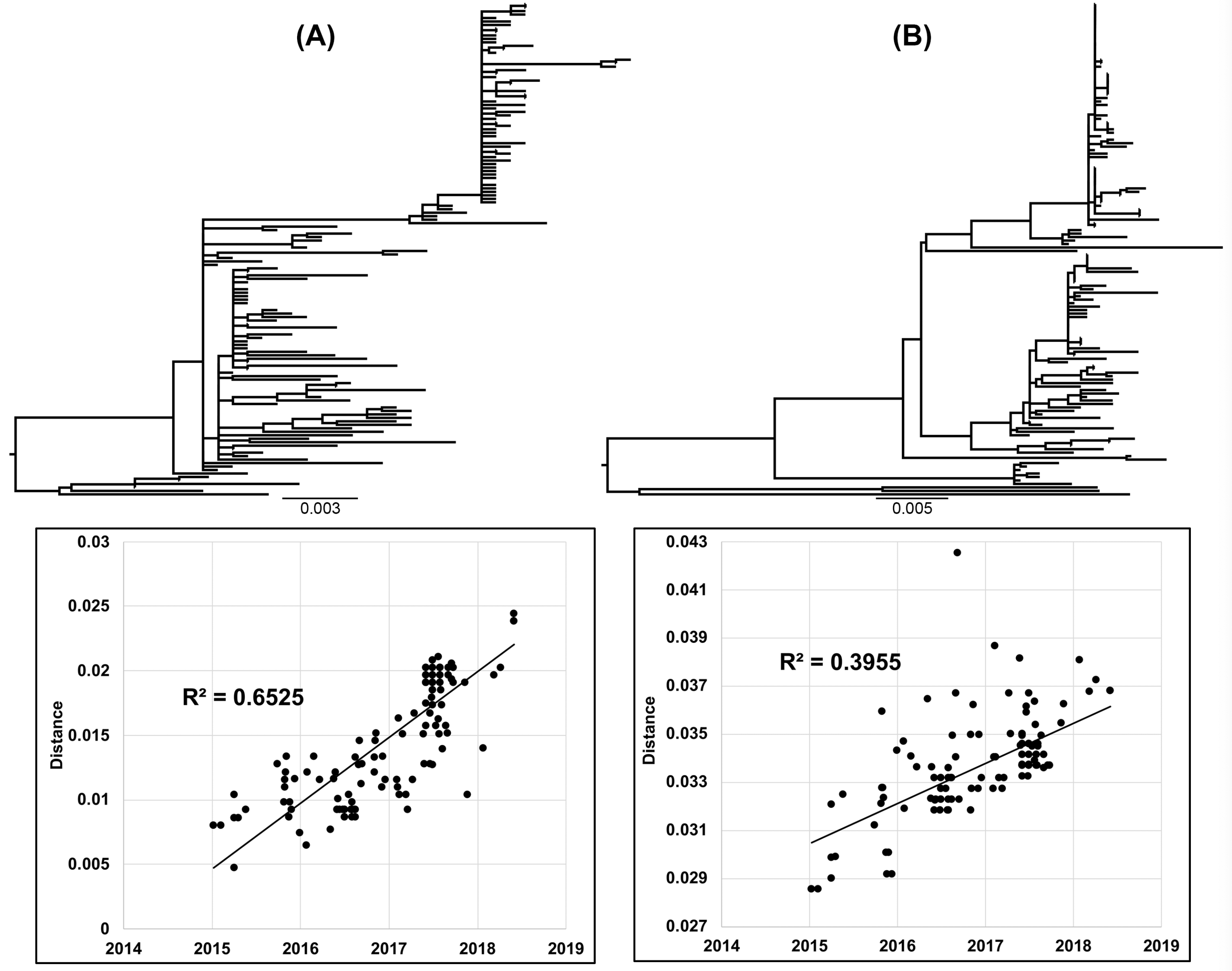


**Supplementary Figure 2.** **Maximum likelihood (ML) phylogeny and root-to-tip genetic distance linear regression of HA and PB2 gene segments of human-like swine influenza virus between January 2015 and June 2018 in the USA.** The trees comprise none identical sequences included in the subsequent BEAST analyses. Scale bar indicate substitution rate per site. (A) represents HA segment (B) represents PB2 segment. (N = 142). Plots at the bottom of each tree represent the root-to-tip genetic distance against sampling time and were estimate using the root-tip-regression analysis feature implemented in TempEst. R^2^s are the estimated correlation coefficients and represent the strength of the association between genetic distances and sampling dates.

Supplementary Tables

**Supplementary Table 1. Summary profile of HA and PB2 gene sequences used for swine influenza phylodynamic analyses (n = 142).**

| ID | Isolate Name | HA Accession | PB2 Accession | State | Date |
| --- | --- | --- | --- | --- | --- |
| 1 | A/swine/Missouri/A01840324/2015 | KP765818 | KT374249 | Missouri | 08-Jan-2015 |
| 2 | A/swine/Missouri/A01840724/2015 | KP901306 | KT356653 | Missouri | 05-Feb-2015 |
| 3 | A/swine/Missouri/A02076058/2015 | KR265517 | KT374259 | Missouri | 01-Apr-2015 |
| 4 | A/swine/Minnesota/A01847237/2015 | KR821159 | KT983800 | Minnesota | 02-Apr-2015 |
| 5 | A/swine/Missouri/A01554378/2015 | KR265506 | KT374239 | Missouri | 02-Apr-2015 |
| 6 | A/swine/Missouri/A01847626/2015 | KR780639 | KT374254 | Missouri | 20-Apr-2015 |
| 7 | A/swine/Iowa/A02077646/2015 | KT164893 | KT983795 | Iowa | 19-May-2015 |
| 8 | A/swine/Iowa/A01944136/2015 | KT965368 | KY401786 | Iowa | 28-Sep-2015 |
| 9 | A/swine/Missouri/A01795668/2015 | KU229918 | KU752478 | Missouri | 26-Oct-2015 |
| 10 | A/swine/Arkansas/A01797067/2015 | KU229909 | KU752394 | Arkansas | 29-Oct-2015 |
| 11 | A/swine/Arkansas/A01797066/2015 | KU229931 | KU752436 | Arkansas | 29-Oct-2015 |
| 12 | A/swine/Ohio/A01795737/2015 | KU229946 | KU752465 | Ohio | 02-Nov-2015 |
| 13 | A/swine/Indiana/A01796207/2015 | KU242623 | KU752415 | Indiana | 03-Nov-2015 |
| 14 | A/swine/Minnesota/A01941094/2015 | KU300983 | KX632012 | Minnesota | 13-Nov-2015 |
| 15 | A/swine/Iowa/A01797426/2015 | KU357017 | KU752491 | Iowa | 17-Nov-2015 |
| 16 | A/swine/Iowa/A01796498/2015 | KU500873 | KX632048 | Iowa | 24-Nov-2015 |
| 17 | A/swine/Iowa/A01727249/2015 | KU513688 | KX772277 | Iowa | 09-Dec-2015 |
| 18 | A/swine/Minnesota/A01941831/2015 | KU598265 | KX772262 | Minnesota | 30-Dec-2015 |
| 19 | A/swine/Minnesota/A01942134/2016 | KU954081 | KX772328 | Minnesota | 26-Jan-2016 |
| 20 | A/swine/Indiana/A01730580/2016 | KU752390 | KX772303 | Indiana | 28-Jan-2016 |
| 21 | A/swine/Iowa/A01732489/2016 | KU942609 | KX772242 | Iowa | 25-Feb-2016 |
| 22 | A/swine/Iowa/A01733965/2016 | KX088430 | KX851865 | Iowa | 21-Mar-2016 |
| 23 | A/swine/Minnesota/A01943420/2016 | KX349974 | KY401873 | Minnesota | 05-May-2016 |
| 24 | A/swine/Iowa/A01775597/2016 | KX380241 | KY401846 | Iowa | 19-May-2016 |
| 25 | A/swine/Iowa/A01775848/2016 | KX394215 | KY115438 | Iowa | 23-May-2016 |
| 26 | A/swine/Ohio/16TOSU3152/2016 | MH283679 | MH283630 | Ohio | 01-Jun-2016 |
| 27 | A/swine/Iowa/16TOSU2772/2016 | MH283554 | MH283639 | Iowa | 01-Jun-2016 |
| 28 | A/swine/Oregon/A01104094/2016 | MF045280 | MF045277 | Oregon | 04-Jun-2016 |
| 29 | A/swine/Iowa/16TOSU2418/2016 | CY219505 | CY219512 | Iowa | 09-Jun-2016 |
| 30 | A/swine/Ohio/16TOSU3220/2016 | CY219747 | CY219754 | Ohio | 25-Jun-2016 |
| 31 | A/swine/Ohio/16TOSU8301/2016 | CY243241 | CY243248 | Ohio | 01-Jul-2016 |
| 32 | A/swine/Ohio/16TOSU8302/2016 | CY243249 | CY243256 | Ohio | 01-Jul-2016 |
| 33 | A/swine/Ohio/16TOSU8254/2016 | CY243209 | CY243216 | Ohio | 01-Jul-2016 |
| 34 | A/swine/Ohio/16TOSU4335/2016 | CY242798 | CY242805 | Ohio | 01-Jul-2016 |
| 35 | A/swine/Iowa/A01778106/2016 | KX761360 | KY401964 | Iowa | 20-Jul-2016 |
| 36 | A/swine/Michigan/A01104095/2016 | KX772285 | KX772282 | Michigan | 28-Jul-2016 |
| 37 | A/swine/Ohio/16TOSU5179/2016 | CY242976 | CY242983 | Ohio | 01-Aug-2016 |
| 38 | A/swine/Indiana/16TOSU4854/2016 | CY242944 | CY242951 | Indiana | 01-Aug-2016 |
| 39 | A/swine/Ohio/16TOSU5399/2016 | CY242992 | CY242999 | Ohio | 01-Aug-2016 |
| 40 | A/swine/Indiana/16TOSU7773/2016 | CY243128 | CY243135 | Indiana | 01-Aug-2016 |
| 41 | A/swine/Kentucky/16TOSU8840/2016 | CY243064 | CY243071 | Kentucky | 01-Aug-2016 |
| 42 | A/swine/Ohio/16TOSU5245/2016 | KX981546 | KX981522 | Ohio | 11-Aug-2016 |
| 43 | A/swine/Ohio/16TOSU8757/2016 | KX981523 | KX981484 | Ohio | 13-Aug-2016 |
| 44 | A/swine/Ohio/16TOSU8764/2016 | KX981552 | KX981501 | Ohio | 13-Aug-2016 |
| 45 | A/swine/Iowa/A01778709/2016 | KX830948 | KY509929 | Iowa | 16-Aug-2016 |
| 46 | A/swine/Iowa/A01781043/2016 | KX908019 | KY678580 | Iowa | 30-Aug-2016 |
| 47 | A/swine/Minnesota/A01781044/2016 | KX928666 | KY509911 | Minnesota | 31-Aug-2016 |
| 48 | A/swine/Minnesota/A01781222/2016 | KX928662 | MF045189 | Minnesota | 07-Sep-2016 |
| 49 | A/swine/Iowa/A01781487/2016 | KX960218 | MF045285 | Iowa | 13-Sep-2016 |
| 50 | A/swine/Kentucky/16TOSU5910/2016 | CY243048 | CY243055 | Kentucky | 01-Nov-2016 |
| 51 | A/swine/Kentucky/16TOSU6069/2016 | CY243056 | CY243063 | Kentucky | 01-Nov-2016 |
| 52 | A/swine/South Dakota/A01678473/2016 | KY174896 | KY938625 | South Dakota | 04-Nov-2016 |
| 53 | A/swine/Missouri/A01668196/2016 | KY328179 | KY938637 | Missouri | 10-Nov-2016 |
| 54 | A/swine/Illinois/A01671222/2016 | KY454604 | MF145399 | Illinois | 02-Dec-2016 |
| 55 | A/swine/Minnesota/A01678475/2016 | KY349113 | MF145451 | Minnesota | 05-Dec-2016 |
| 56 | A/swine/Missouri/A01671485/2016 | KY465585 | MF145474 | Missouri | 15-Dec-2016 |
| 57 | A/swine/Missouri/A01672702/2017 | KY744622 | MF522056 | Missouri | 03-Feb-2017 |
| 58 | A/swine/Oklahoma/A01932035/2017 | KY766088 | MF522026 | Oklahoma | 06-Feb-2017 |
| 59 | A/swine/Illinois/0TM110/2017 | MF973440 | MF973439 | Illinois | 13-Feb-2017 |
| 60 | A/swine/Iowa/A01932578/2017 | KY888281 | MF522014 | Iowa | 27-Feb-2017 |
| 61 | A/swine/Missouri/A01667102/2017 | KY941143 | MF801484 | Missouri | 13-Mar-2017 |
| 62 | A/swine/Ohio/A02214230/2017 | KY995605 | MF872643 | Ohio | 20-Mar-2017 |
| 63 | A/swine/Iowa/A02214497/2017 | MF159358 | MF973308 | Iowa | 09-Apr-2017 |
| 64 | A/swine/Illinois/A02214843/2017 | MF150346 | MF973326 | Illinois | 14-Apr-2017 |
| 65 | A/swine/North Carolina/A02216061/2017 | MF289439 | MG193897 | North Carolina | 22-May-2017 |
| 66 | A/swine/Minnesota/A02216466/2017 | MF375245 | MG193825 | Minnesota | 25-May-2017 |
| 67 | A/swine/Indiana/17TOSU2840/2017 | MK535326 | MK535333 | Indiana | 01-Jun-2017 |
| 68 | A/swine/Indiana/17TOSU1135/2017 | MK109675 | MK109682 | Indiana | 01-Jun-2017 |
| 69 | A/swine/Indiana/17TOSU1123/2017 | MK109699 | MK109706 | Indiana | 01-Jun-2017 |
| 70 | A/swine/Ohio/17TOSU5579/2017 | MK536482 | MK536489 | Ohio | 01-Jun-2017 |
| 71 | A/swine/Ohio/17TOSU5564/2017 | MK536457 | MK536465 | Ohio | 01-Jun-2017 |
| 72 | A/swine/Ohio/17TOSU5637/2017 | MK536562 | MK536569 | Ohio | 01-Jun-2017 |
| 73 | A/swine/Ohio/17TOSU5554/2017 | MK536407 | MK536414 | Ohio | 01-Jun-2017 |
| 74 | A/swine/Ohio/17TOSU5114/2017 | MK536215 | MK536222 | Ohio | 01-Jun-2017 |
| 75 | A/swine/Ohio/17TOSU2596/2017 | MK109475 | MK109482 | Ohio | 01-Jun-2017 |
| 76 | A/swine/Indiana/17TOSU2835/2017 | MK109251 | MK109258 | Indiana | 01-Jun-2017 |
| 77 | A/swine/Ohio/17TOSU5521/2017 | MK536383 | MK536390 | Ohio | 01-Jun-2017 |
| 78 | A/swine/Indiana/17TOSU3748/2017 | MK535398 | MK535405 | Indiana | 01-Jun-2017 |
| 79 | A/swine/Ohio/17TOSU5172/2017 | MK536327 | MK536334 | Ohio | 01-Jun-2017 |
| 80 | A/swine/Ohio/17TOSU5506/2017 | MK536367 | MK536374 | Ohio | 01-Jun-2017 |
| 81 | A/swine/Indiana/17TOSU2865/2017 | MK535366 | MK535373 | Indiana | 01-Jun-2017 |
| 82 | A/swine/Indiana/17TOSU2838/2017 | MK535310 | MK535317 | Indiana | 01-Jun-2017 |
| 83 | A/swine/Illinois/17TOSU5908/2017 | MK535270 | MK535277 | Illinois | 01-Jun-2017 |
| 84 | A/swine/Ohio/17TOSU5580/2017 | MK536490 | MK536497 | Ohio | 01-Jun-2017 |
| 85 | A/swine/Indiana/17TOSU1066/2017 | MK109560 | MK109567 | Indiana | 01-Jun-2017 |
| 86 | A/swine/Ohio/17TOSU5582/2017 | MK536506 | MK536513 | Ohio | 01-Jun-2017 |
| 87 | A/swine/Indiana/17TOSU2855/2017 | MK535358 | MK535365 | Indiana | 01-Jun-2017 |
| 88 | A/swine/Iowa/17TOSU3483/2017 | MK535759 | MK535766 | Iowa | 01-Jun-2017 |
| 89 | A/swine/Indiana/17TOSU1056/2017 | MK109052 | MK109059 | Indiana | 01-Jun-2017 |
| 90 | A/swine/Minnesota/A01785267/2017 | MF425640 | MG521066 | Minnesota | 01-Jun-2017 |
| 91 | A/swine/Iowa/A02218163/2017 | MF613964 | MG521054 | Iowa | 21-Jun-2017 |
| 92 | A/swine/Pennsylvania/A02218184/2017 | MF673427 | MG521090 | Pennsylvania | 21-Jun-2017 |
| 93 | A/swine/Iowa/A02218173/2017 | MF582530 | MG521096 | Iowa | 27-Jun-2017 |
| 94 | A/swine/Kentucky/17TOSU6438/2017 | MK535975 | MK535982 | Kentucky | 01-Jul-2017 |
| 95 | A/swine/Indiana/17TOSU1724/2017 | MK109173 | MK109181 | Indiana | 01-Jul-2017 |
| 96 | A/swine/Indiana/17TOSU1347/2017 | MK109633 | MK109640 | Indiana | 01-Jul-2017 |
| 97 | A/swine/Kentucky/17TOSU6521/2017 | MK535999 | MK536006 | Kentucky | 01-Jul-2017 |
| 98 | A/swine/Indiana/17TOSU1568/2017 | MK109617 | MK109624 | Indiana | 01-Jul-2017 |
| 99 | A/swine/Kentucky/17TOSU6368/2017 | MK535935 | MK535942 | Kentucky | 01-Jul-2017 |
| 100 | A/swine/Ohio/17TOSU1420/2017 | MK109100 | MK109107 | Ohio | 01-Jul-2017 |
| 101 | A/swine/Kentucky/17TOSU6329/2017 | MK535927 | MK535934 | Kentucky | 01-Jul-2017 |
| 102 | A/swine/Kentucky/17TOSU6581/2017 | MK536039 | MK536046 | Kentucky | 01-Jul-2017 |
| 103 | A/swine/Kentucky/17TOSU6417/2017 | MK535943 | MK535950 | Kentucky | 01-Jul-2017 |
| 104 | A/swine/Indiana/17TOSU1731/2017 | MK109511 | MK109518 | Indiana | 01-Jul-2017 |
| 105 | A/swine/Ohio/17TOSU1404/2017 | MK109609 | MK109616 | Ohio | 01-Jul-2017 |
| 106 | A/swine/Indiana/17TOSU1833/2017 | MH255633 | MH255650 | Indiana | 01-Jul-2017 |
| 107 | A/swine/Illinois/17TOSU5489/2017 | MK535198 | MK535205 | Illinois | 01-Jul-2017 |
| 108 | A/swine/Kentucky/17TOSU6274/2017 | MK535911 | MK535918 | Kentucky | 01-Jul-2017 |
| 109 | A/swine/Ohio/17TOSU4304/2017 | MK536095 | MK536102 | Ohio | 01-Jul-2017 |
| 110 | A/swine/Ohio/17TOSU1801/2017 | MK109156 | MK109163 | Ohio | 01-Jul-2017 |
| 111 | A/swine/Iowa/A02218427/2017 | MF623902 | MG662559 | Iowa | 13-Jul-2017 |
| 112 | A/swine/Ohio/A02218759/2017 | MF784417 | MG662589 | Ohio | 25-Jul-2017 |
| 113 | A/swine/Illinois/A02218757/2017 | MF784413 | MG662631 | Illinois | 25-Jul-2017 |
| 114 | A/swine/Iowa/A02218753/2017 | MF784423 | MG662683 | Iowa | 26-Jul-2017 |
| 115 | A/swine/Michigan/17TOSU4222/2017 | MK109427 | MK109434 | Michigan | 01-Aug-2017 |
| 116 | A/swine/Ohio/17TOSU4118/2017 | MK109491 | MK109498 | Ohio | 01-Aug-2017 |
| 117 | A/swine/Ohio/17TOSU4107/2017 | MK109028 | MK109035 | Ohio | 01-Aug-2017 |
| 118 | A/swine/West Virginia/17TOSU4485/2017 | MK109371 | MK109378 | West Virginia | 01-Aug-2017 |
| 119 | A/swine/Ohio/17TOSU7274/2017 | MK109715 | MK109722 | Ohio | 01-Aug-2017 |
| 120 | A/swine/Iowa/17TOSU7821/2017 | MK109307 | MK109314 | Iowa | 01-Aug-2017 |
| 121 | A/swine/Indiana/17TOSU7478/2017 | MK109551 | MK109558 | Indiana | 01-Aug-2017 |
| 122 | A/swine/Ohio/17TOSU4320/2017 | MK109132 | MK109139 | Ohio | 01-Aug-2017 |
| 123 | A/swine/Ohio/A01354314/2017 | MF801511 | MF801508 | Ohio | 04-Aug-2017 |
| 124 | A/swine/Iowa/A02218762/2017 | MF784427 | MG784664 | Iowa | 07-Aug-2017 |
| 125 | A/swine/Iowa/A02219544/2017 | MF920410 | MG784640 | Iowa | 08-Aug-2017 |
| 126 | A/swine/Iowa/A02219788/2017 | MF962624 | MG784586 | Iowa | 22-Aug-2017 |
| 127 | A/swine/Minnesota/A02221495/2017 | MG198957 | MG784598 | Minnesota | 29-Aug-2017 |
| 128 | A/swine/Ohio/17TOSU4791/2017 | MK109747 | MK109754 | Ohio | 01-Sep-2017 |
| 129 | A/swine/Ohio/17TOSU4798/2017 | MK109068 | MK109075 | Ohio | 01-Sep-2017 |
| 130 | A/swine/Missouri/A01932047/2017 | KY766072 | MF664395 | Missouri | 09-Feb-2017 |
| 131 | A/swine/Maryland/A01764002/2017 | MG193840 | MG193837 | Maryland | 17-Sep-2017 |
| 132 | A/swine/Maryland/A01764005/2017 | MG193878 | MG193875 | Maryland | 17-Sep-2017 |
| 133 | A/swine/Maryland/A01764022/2017 | MG193886 | MG193883 | Maryland | 23-Sep-2017 |
| 134 | A/swine/Maryland/A01764024/2017 | MG193802 | MG193799 | Maryland | 23-Sep-2017 |
| 135 | A/swine/Ohio/OH-17-25426/2017 | MG982492 | MG982498 | Ohio | 10-Nov-2017 |
| 136 | A/swine/Ohio/OH-17-26330/2017 | MG982507 | MG982514 | Ohio | 22-Nov-2017 |
| 137 | A/swine/Missouri/A02200041/2018 | MG983210 | MG983207 | Missouri | 25-Jan-2018 |
| 138 | A/swine/Ohio/OH-18-5253/2018 | MH234399 | MH234406 | Ohio | 09-Mar-2018 |
| 139 | A/swine/Ohio/OH-18-7969/2018 | MH234447 | MH234454 | Ohio | 06-Apr-2018 |
| 140 | A/swine/Indiana/18TOSU3605/2018 | MH791962 | MH791951 | Indiana | 01-Jun-2018 |
| 141 | A/swine/Indiana/18TOSU3614/2018 | MH791956 | MH791964 | Indiana | 01-Jun-2018 |
| 142 | A/swine/Indiana/18TOSU3611/2018 | MH791959 | MH791949 | Indiana | 01-Jun-2018 |

**Supplementary Table 2. Temporal and geographical summary profile of HA and PB2 gene sequences used for swine influenza phylodynamic analyses (n = 142).**

|  | *2015* | *2016* | *2017* | *2018* | **Total** |
| --- | --- | --- | --- | --- | --- |
| Arkansas | 2 | - | - | - | 2 |
| Illinois | - | 1 | 5 | - | 6 |
| Indiana | 1 | 3 | 16 | 3 | 23 |
| Iowa | 5 | 10 | 11 |  | 26 |
| Kentucky | - | 3 | 7 | - | 10 |
| Maryland | - |  | 4 | - | 4 |
| Michigan | - | 1 | 1 | - | 2 |
| Minnesota | 3 | 5 | 3 | - | 11 |
| Missouri | 6 | 2 | 3 | 1 | 12 |
| North Carolina | - | - | 1 |  | 1 |
| Ohio | 1 | 11 | 25 | 2 | 39 |
| Oklahoma | - | - | 1 |  | 1 |
| Oregon | - | 1 | - | - | 1 |
| Pennsylvania | - | - | 1 | - | 1 |
| South Dakota | - | 1 | - | - | 1 |
| Wisconsin | - | - | 1 | - | 1 |
| West Virginia | - | - | 1 | - | 1 |
| **Total** | 18 | 38 | 80 | 6 | 142 |

**Supplementary Table 3. Bayes factor (BF) comparisons of HA gene segment asymmetric discrete phylogeographic models using the path-sampling (PS) and the stepping-stone (SS) marginal likelihood estimators.** PS BFs are on the upper off-diagonal of the table, while SS BFs are on the lower off-diagonal of the table. Best fitting models have been boldfaced.

|  |  |  |  | Bayes Factor |  |  |  |  |
| --- | --- | --- | --- | --- | --- | --- | --- | --- |
| Model | UCED+CP | UCED+EG | UCED+EGx | UCED+SG | UCLN+CP | UCLN+EG | UCLN+EGx | UCLN+SG |
| UCEDa+CPb | –– | 3 | 1 | -93 | -45 | -49 | -49 | -237 |
| UCED+EGc | -5 | –– | -2 | -96 | -48 | -52 | -52 | -240 |
| UCED+EGxd | -1 | 3 | –– | -94 | -46 | -50 | -50 | -238 |
| UCED+SGf | 96 | 101 | 97 | –– | 48 | 45 | 44 | -144 |
| UCLNg+CP | 45 | 50 | 47 | -50.6 | –– | -4 | -4 | -192 |
| UCLN+EG | 48 | 52 | 49 | -48 | 2 | –– | -1 | -189 |
| UCLN+EGx | 50 | 54 | 51 | -47 | 4 | 2 | –– | -188 |
| **UCLN+SG** | **240** | **241** | **241** | **144** | **195** | **193** | **191** | **––** |

^a^Uncorrelated relaxed clock with exponential distribution

^b^Constant population size coalescent model

^c^Expansion population size coalescent model

^d^Exponential population size coalescent model

^f^Bayesian skylgrid coalescent model

^g^Uncorrelated relaxed clock with log-normal distribution

**Supplementary Table 4. Bayes factor (BF) comparisons of HA gene segment symmetric discrete phylogeographic models using the path-sampling (PS) and the stepping-stone (SS) marginal likelihood estimators.** PS BFs are on the upper off-diagonal of the table, while SS BFs are on the lower off-diagonal of the table. Best fitting models have been boldfaced.

|  |  |  |  | Bayes Factor |  |  |  |  |
| --- | --- | --- | --- | --- | --- | --- | --- | --- |
| Model | UCED+CP | UCED+EG | UCED+EGx | UCED+SG | UCLN+CP | UCLN+EG | UCLN+EGx | UCLN+SG |
| UCED^a^+CP^b^ | –– | -6 | -4 | -42 | -4 | -10 | -6 | -72 |
| UCED+EG^c^ | 8 | –– | 2 | -36 | 2 | -4 | 0 | -66 |
| UCED+EGx^d^ | 4 | -4 | –– | -38 | 0 | -6 | -2 | -68 |
| UCED+SG^f^ | 46 | 38 | 42 | –– | 38 | 32 | 36 | -30 |
| UCLN^g^+CP | 4 | -4 | 0 | -42 | –– | -6 | -2 | -68 |
| UCLN+EG | 10 | 2 | 6 | -36 | 6 | –– | 4 | -62 |
| UCLN+EGx | 6 | -2 | 2 | -40 | 2 | -4 | –– | -66 |
| **UCLN+SG** | **74** | **70** | **70** | **28** | **70** | **64** | **68** | **––** |

^a^Uncorrelated relaxed clock with exponential distribution

^b^Constant population size coalescent model

^c^Expansion population size coalescent model

^d^Exponential population size coalescent model

^f^Bayesian skylgrid coalescent model

^g^Uncorrelated relaxed clock with log-normal distribution

**Supplementary Table 5. Bayes factor (BF) comparisons of PB2 gene segment asymmetric discrete phylogeographic models using the path-sampling (PS) and the stepping-stone (SS) marginal likelihood estimators.** PS BFs are on the upper off-diagonal of the table, while SS BFs are on the lower off-diagonal of the table. Best fitting models have been boldfaced.

|  |  |  |  | Bayes Factor |  |  |  |  |
| --- | --- | --- | --- | --- | --- | --- | --- | --- |
| Model | UCED+CP | UCED+EG | UCED+EGx | UCED+SG | UCLN+CP | UCLN+EG | UCLN+EGx | UCLN+SG |
| UCED^a^+CP^b^ | –– | -14 | -2 | -28 | 10 | 14 | 8 | -16 |
| UCED+EG^c^ | 14 | –– | 12 | -14 | 24 | 28 | 22 | -2 |
| UCED+EGx^d^ | 0 | -14 | –– | -26 | 12 | 16 | 10 | -14 |
| **UCED+SG^f^** | **30** | **16** | **30** | **––** | **38** | **42** | **36** | **12** |
| UCLN^g^+CP | -12 | -26 | -12 | -42 | –– | 4 | -2 | -26 |
| UCLN+EG | -14 | -28 | -14 | -44 | -2 | –– | -6 | -30 |
| UCLN+EGx | -6 | -20 | -6 | -36 | 6 | 8 | –– | -24 |
| UCLN+SG | 20 | 20 | 20 | -10 | 32 | 34 | 26 | –– |

^a^Uncorrelated relaxed clock with exponential distribution

^b^Constant population size coalescent model

^c^Expansion population size coalescent model

^d^Exponential population size coalescent model

^f^Bayesian skylgrid coalescent model

^g^Uncorrelated relaxed clock with log-normal distribution

**Supplementary Table 6. Bayes factor (BF) comparisons of PB2 gene segment symmetric discrete phylogeographic models using the path-sampling (PS) and the stepping-stone (SS) marginal likelihood estimators.** PS BFs are on the upper off-diagonal of the table, while SS BFs are on the lower off-diagonal of the table. Best fitting models have been boldfaced.

|  |  |  |  | Bayes Factor |  |  |  |  |
| --- | --- | --- | --- | --- | --- | --- | --- | --- |
| Model | UCED+CP | UCED+EG | UCED+EGx | UCED+SG | UCLN+CP | UCLN+EG | UCLN+EGx | UCLN+SG |
| UCED^a^+CP^b^ | –– | 12 | 8 | -12 | 12 | 16 | 10 | -4 |
| UCED+EG^c^ | -12 | –– | -4 | -24 | 0 | 4 | -2 | -16 |
| UCED+EGx^d^ | -8 | 4 | –– | -20 | 4 | 8 | 2 | -12 |
| **UCED+SG^f^** | **14** | **26** | **22** | **––** | **24** | **28** | **22** | **8** |
| UCLN^g^+CP | -12 | 0 | -4 | -26 | –– | 4 | -2 | -16 |
| UCLN+EG | -18 | -6 | -10 | -32 | -6 | –– | -6 | -20 |
| UCLN+EGx | -12 | 0 | -4 | -26 | 0 | 6 | –– | -14 |
| UCLN+SG | 4 | 12 | 12 | -10 | 16 | 22 | 16 | –– |

^a^Uncorrelated relaxed clock with exponential distribution

^b^Constant population size coalescent model

^c^Expansion population size coalescent model

^d^Exponential population size coalescent model

^f^Bayesian skylgrid coalescent model

^g^Uncorrelated relaxed clock with log-normal distribution
